# Adaptive Gating and Focal Debiasing for robust few-shot retinal disease classification

**DOI:** 10.3389/frai.2026.1825132

**Published:** 2026-05-26

**Authors:** Stewart Muchuchuti, Serestina Viriri

**Affiliations:** Discipline of Computer Science, School of Agriculture and Science, University of KwaZulu-Natal, Durban, South Africa

**Keywords:** attention mechanism, class imbalance, computer-aided diagnosis, deep learning, dynamic gating, few-shot learning, focal loss, retinal imaging

## Abstract

Hybrid attention few-shot models are promising for automated retinal disease classification in data-scarce settings, yet two issues persist: static fusion of global and local features, and weak performance on minority classes. We propose Adaptive Gating and Focal Debiasing (AGFD), which adds a Dynamic Attention Gating (DAG) module that learns input-specific weights for global and local attention branches, and replaces cross-entropy with focal loss to shift learning toward hard, underrepresented cases. On ODIR-5K under episodic evaluation, AGFD improves over a strong hybrid-attention baseline across all shot settings, reaching 78.7% accuracy and 76.9% macro-F1 in the 5-shot setting, and 83.2% accuracy at 10-shot. Minority classes benefit most, with +11–15 percentage-point F1 gains for Glaucoma, Cataract, Hypertension, and Other. Gate analysis shows higher global weighting for widespread conditions and higher local weighting for lesion-driven diseases. Coupling adaptive fusion with a debiased objective improves overall accuracy and reliability on underrepresented classes, a step toward more clinically useful screening.

## 1 Introduction

The early and accurate diagnosis of retinal diseases, such as diabetic retinopathy, glaucoma, and age-related macular degeneration, is essential to prevent irreversible vision loss, a condition that affects millions around the world (Litjens et al., 2017; Hassan et al., 2021). Deep learning–powered automated diagnostic systems have become important tools for ophthalmologists by providing rapid and reliable disease classification (Muchuchuti and Viriri, 2023). However, the development of robust models faces a significant hurdle: the scarcity of labeled data for many pathologies. This is a common constraint in medical imaging, where expert annotation is both costly and time-consuming (Burlina et al., 2020).

Few-shot learning (FSL) has been identified as a promising paradigm to address data scarcity, enabling models to generalize from a small number of annotated examples (Snell et al., 2017). Recent advancements have explored transformer-based architectures (Yu et al., 2022) and meta-learning paradigms to improve generalization. However, in medical imaging, where diagnostic information resides at multiple scales, combining the inductive biases of Convolutional Neural Networks (CNNs) with attention mechanisms offers a compelling balance of computational efficiency and performance. Our previous work (Muchuchuti and Viriri, 2026) demonstrated the value of a hybrid framework combining a *sparse cross-attention* module for global feature extraction with a *lightweight multi-attention* module for capturing fine-grained local details.

Despite the promise of hybrid attention, existing approaches often rely on static feature fusion mechanisms; they combine global and local attention outputs through simple concatenation, treating all images uniformly. This fixed strategy is suboptimal. Some retinal diseases are characterized by widespread structural changes (e.g., Myopia), for which global features are more informative, while others are defined by small localized lesions (e.g., early-stage diabetic retinopathy), where local features are critical. A significant methodological advancement is required to transition from static fusion to a dynamic, input-adaptive architecture.

Furthermore, the performance of FSL models suffers significantly in underrepresented disease classes due to the severe class imbalance inherent in real-world medical datasets such as ODIR-5K. Models trained on imbalanced datasets tend to develop a bias toward majority classes, resulting in markedly lower F1 scores for dataset-specific minority classes such as hypertension (H) and other abnormalities (O). As highlighted by recent literature, this missingness or imbalance is often informative rather than random, and specialized strategies are required to handle limited or imperfect supervision (Wu et al., 2026; Li et al., 2026). This diagnostic disparity remains a major barrier to clinical deployment, where reliable detection of all conditions, regardless of prevalence, is essential for patient safety.

To overcome these limitations, this article proposes a novel framework: Adaptive Gating and Focal Debiasing (AGFD). This work introduces two main contributions to create a more reliable, equitable, and intelligent diagnostic model.

1. A Dynamic Attention Gating (DAG) mechanism. A lightweight, learnable gating module is introduced which adaptively balances the influence of the global and local attention branches. This allows the model to learn an optimal input-specific feature fusion strategy, prioritizing the most relevant feature scale for the pathology being analyzed.
2. A debiased training objective using Focal Loss. To directly address the class imbalance problem, the standard cross-entropy objective is replaced with a Focal Loss function (Lin et al., 2017). This modification forces the model to focus its training efforts on hard-to-classify examples, which are predominantly from minority classes, thereby improving diagnostic accuracy for underrepresented conditions.

Through comprehensive experiments on the ODIR-5K dataset and external benchmarks, it is demonstrated that AGFD achieves significant improvements in both overall accuracy and minority class performance, while maintaining robust out-of-distribution (OOD) detection capabilities.

The remainder of this paper is organized as follows. Section 2 reviews related work on FSL, attention mechanisms, and handling of class imbalances. Section 3 presents the proposed AGFD framework, including the DAG module and focal loss integration. Section 4 describes the experimental setup and datasets. Section 5 reports comprehensive results, including ablation studies and qualitative analysis. Finally, Section 6 discusses the findings, limitations, and future directions.

## 2 Related work

This section reviews the key research areas that form the foundation of the proposed work. First, recent advances in FSL are discussed, including transformer-based and meta-learning approaches. Second, the role of attention mechanisms and multi-scale feature fusion in medical imaging is examined. Third, the literature on dynamic and adaptive neural architectures is explored, which informs the gating mechanism. Finally, common techniques for addressing class imbalance are surveyed, justifying the choice of Focal Loss.

### 2.1 Recent advances in few-shot learning

In recent years, the field of FSL has seen rapid advancements, particularly with the advent of transformer-based architectures and large-scale pre-trained models. MetaFormer approaches (Yu et al., 2022) have demonstrated the effectiveness of combining meta-learning with transformer architectures for FSL tasks, achieving strong performance through learned initialization strategies and attention-based feature extraction. Similarly, vision-language models such as Contrastive Language-Image Pre-training (CLIP) have been successfully adapted for few-shot classification through methods like CLIP-FSL (Feng et al., 2024), which leverage pre-trained semantic knowledge to improve generalization from limited examples. Hybrid architectures combining Vision Transformers (ViT) and Convolutional Neural Networks (CNN) (Kim et al., 2025) have also emerged as a promising direction, exploiting the complementary strengths of both paradigms: CNNs excel at capturing local spatial patterns through inductive biases, while transformers effectively model long-range dependencies through self-attention.

While these methods have pushed the boundaries of FSL in natural image domains, they often do not explicitly address the unique challenges of medical imaging. Medical images require robust performance on minority classes, interpretability for clinical trust, and the ability to handle domain shifts across different imaging devices and populations. Recent theoretical work by Wu et al. (2026) demonstrates that in semi-supervised and limited-label learning, missingness is often informative, and modeling this structure can enhance inference when labeled data are sparse. Furthermore, Li et al. (2026) highlight the importance of training set refinement and prototype calibration for generalized few-shot classification under class imbalance. The AGFD framework is specifically designed to address these challenges through its DAG mechanism, which provides input-adaptive feature fusion, and its focal debiasing objective, which directly targets the class imbalance problem prevalent in medical datasets. Unlike general-purpose FSL methods that prioritize overall accuracy, AGFD emphasizes equitable performance across all disease classes, making it more suitable for clinical deployment.

### 2.2 Attention mechanisms and multi-scale feature fusion

Attention mechanisms have become a cornerstone of modern deep learning models for medical image analysis, enabling them to focus on the most salient features while suppressing irrelevant information (Schlemper et al., 2019). In the context of retinal imaging, attention is particularly effective as it can mimic the diagnostic process of an ophthalmologist, who selectively examines specific regions of the fundus. Various forms of attention have been explored. For instance, Woo et al. (2018) proposed the Convolutional Block Attention Module (CBAM), which sequentially infers attention maps along both channel and spatial dimensions to refine features. Schlemper et al. (2019) introduced Attention Gated Networks, which use gating signals to control feature importance in convolutional neural networks (CNNs) for medical image segmentation, demonstrating improved performance by focusing on relevant anatomical structures.

A critical aspect of retinal image analysis is the need to process information at multiple scales. Pathological indicators can range from large-scale variations in the vascular tree to tiny, localized microaneurysms. Architectures such as U-Net (Ronneberger et al., 2015) have been highly effective in medical imaging because their encoder–decoder structure with skip connections naturally fuses features across different scales. Addressing this multi-scale challenge requires capturing both global, long-range dependencies and fine-grained, localized features. While previous hybrid attention frameworks (Muchuchuti and Viriri, 2026) have combined sparse cross-attention and lightweight multi-attention modules, they have typically relied on a static fusion of the two attention pathways through simple concatenation. This presents a notable limitation, as it cannot adapt to the varying spatial scales of different pathologies. The present work addresses this by introducing a dynamic layer that learns to weight these pathways adaptively, a concept elaborated in the next subsection.

### 2.3 Dynamic and adaptive neural architectures

The architecture of most deep learning models, such as the ResNet backbone (He et al., 2016) used in the baseline model, is static. The same computational path and set of parameters are used for every input. However, a growing body of research explores dynamic architectures that can adapt their structure or computational graph based on the input data. A prominent example is the Mixture-of-Experts (MoE) paradigm, first introduced by Jacobs et al. (1991) and later scaled up for deep networks by Shazeer et al. (2017). In an MoE layer, a “gating network” learns to route each input token to a subset of “expert” sub-networks. This allows the model to specialize its components for different types of data, increasing its capacity without a proportional increase in computational cost for any single input.

This principle of using a gating network to control information flow is central to the present work. While MoE models typically route data to different experts, the proposed approach adapts the concept to dynamically weigh the outputs of different feature extractors. This idea of adaptive feature modulation is also seen in other contexts. For instance, Batch Normalization (Wu and He, 2018) can be viewed as a simple form of adaptive behavior, as it normalizes features based on batch statistics. More complex gating mechanisms have been used for multi-modal fusion, such as in the Cross-Modal Transformer (CMT) (Guo et al., 2022), which uses gates to effectively fuse features from CNN and transformer branches. Inspired by this literature, the DAG mechanism is designed as a lightweight, learnable module that explicitly learns the optimal balance between global and local feature representations for retinal disease classification, making the model's architecture adaptive.

### 2.4 Class imbalance in medical diagnostics

Class imbalance is a pervasive challenge in medical imaging, where the prevalence of healthy cases or common diseases far outweighs that of minority classes. When trained on such imbalanced datasets, standard deep learning models tend to develop a bias toward the majority classes, resulting in poor sensitivity for detecting minority but clinically important conditions (Burlina et al., 2020). This issue was also noted in our previous work (Muchuchuti and Viriri, 2026), where the baseline model struggled to identify the underrepresented Hypertension (H) and Other (O) classes.

The challenge of data scarcity is fundamentally linked to the sample size required to achieve reliable classification performance. As demonstrated by (Salazar et al. 2022), the relationship between training set size and classification error can be analyzed through learning curves, which provide theoretical bounds on the expected performance given a finite number of labeled samples. Data augmentation is a common strategy to artificially increase the effective sample size (Yun et al., 2019), but it cannot fully compensate for the lack of true diversity in very small datasets. This motivates the need for FSL approaches that can extract maximum information from limited examples while maintaining robust generalization.

A variety of strategies have been developed to counteract the class imbalance problem. Data-level approaches involve resampling the dataset, for example, by over-sampling the minority class using techniques like the Synthetic Minority Over-sampling Technique (SMOTE) (Chawla et al., 2002), or by under-sampling the majority class. However, these methods can lead to overfitting or the loss of important information. Recent advances in generative models have led to more sophisticated oversampling techniques. Notably, Generative Adversarial Network Synthesis for Oversampling (GANSO) (Salazar et al., 2021) has been shown to outperform SMOTE in scenarios with very small training sets, particularly in biomedical applications such as functional magnetic resonance imaging (fMRI). GANSO uses a GAN-based approach combined with Markov Random Fields to generate more realistic synthetic samples that better capture the underlying data distribution. While such generative approaches are promising, they introduce additional complexity and computational cost, and their effectiveness in few-shot episodic training remains an open question (Wang et al., 2020).

Algorithm-level approaches, which modify the learning process itself, are often more effective and computationally efficient. These can be broadly categorized into re-weighting and re-margining strategies. Cost-sensitive learning, a form of re-weighting, assigns a higher misclassification penalty to minority classes. Cui et al. (2019) proposed a class-balanced loss based on the “effective number of samples,” which weights the loss inversely proportional to the number of samples in each class. The Focal Loss, introduced by Lin et al. (2017), is a more advanced re-weighting technique that dynamically adjusts the weight based on classification confidence. It down-weights the contribution of easy-to-classify, majority-class examples, forcing the model to concentrate on “hard” examples, which are often the minority class samples. Other methods, like Equalization Loss (Tan et al., 2021), focus on re-margining by penalizing the model for focusing too much on frequent negative classes. Given its proven effectiveness, elegant formulation, direct focus on hard examples, and computational efficiency, Focal Loss is adopted as the core of the debiased training objective.

## 3 Proposed methodology

The Adaptive Gating and Focal Debiasing (AGFD) framework extends the hybrid attention-based architecture described in our previous work (Muchuchuti and Viriri, 2026). It introduces two key enhancements: (i) a DAG module that adaptively fuses global and local attention features, and (ii) a composite training objective that replaces cross-entropy with focal loss and incorporates a prototype distance margin loss to improve OOD robustness. While previous work has examined static concatenation of branch features and cost-sensitive cross-entropy reweighting (Gu et al., 2025; Jin et al., 2024), these strategies remain limited: concatenation provides no input-adaptive weighting, and cost-sensitive reweighting focuses on class frequency rather than directly addressing hard examples within episodic FSL.

### 3.1 Framework overview

**Figure 1:**
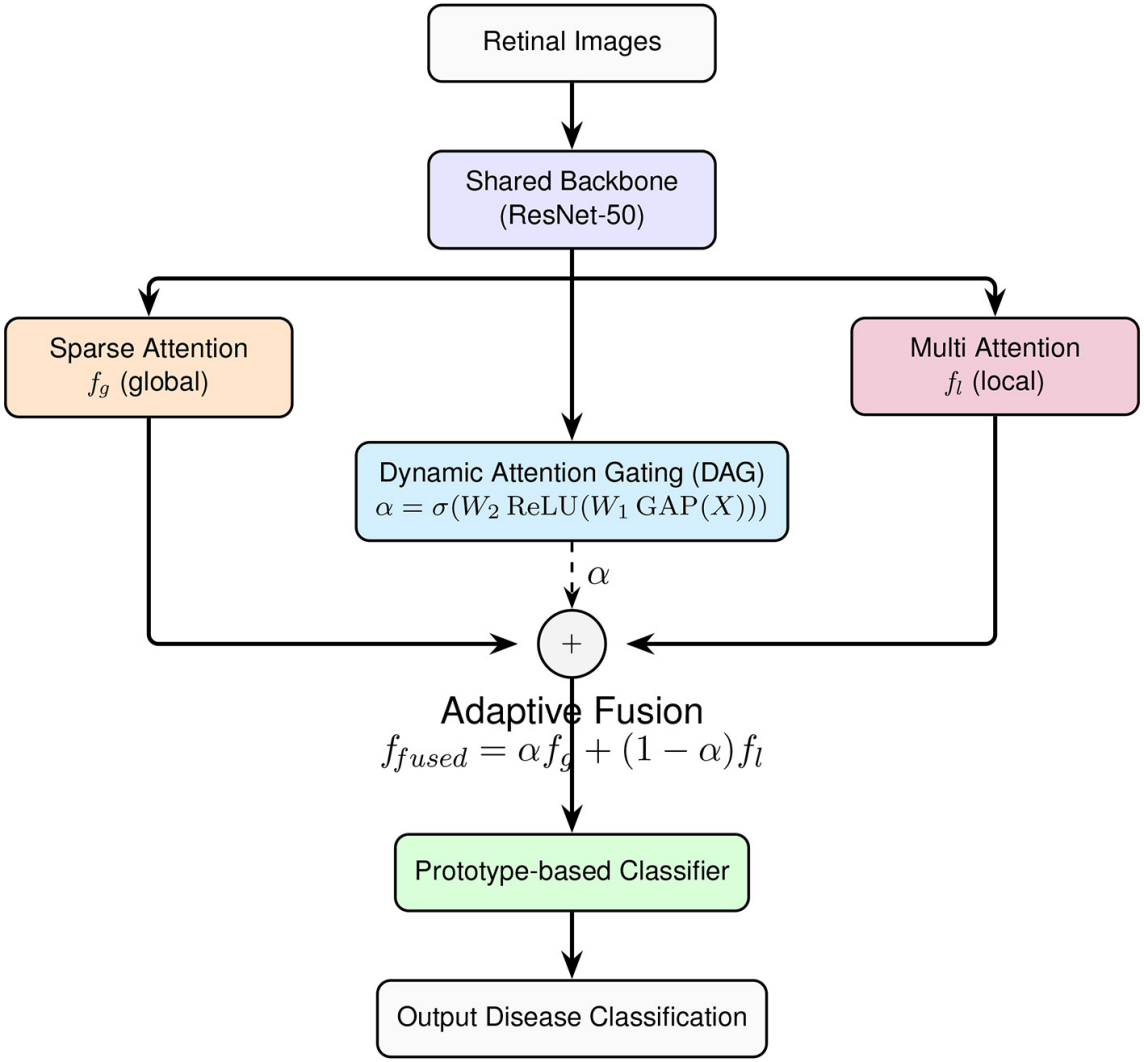
Proposed AGFD framework.

The general architecture of AGFD is shown in Figure 1. A ResNet-50 backbone produces shared features that are forwarded to two parallel attention branches: (i) a sparse cross-attention module for capturing global contextual information, and (ii) a lightweight multi-attention module for fine-grained local details. In contrast to the baseline described in our previous work (Muchuchuti and Viriri, 2026), these features are not fused via simple concatenation. Instead, the DAG module computes a dynamic scalar weight α to balance the contributions of the two branches. The resulting fused embedding is subsequently passed to a prototype-based classifier.

### 3.2 Dynamic Attention Gating (DAG) module

**Figure 2:**
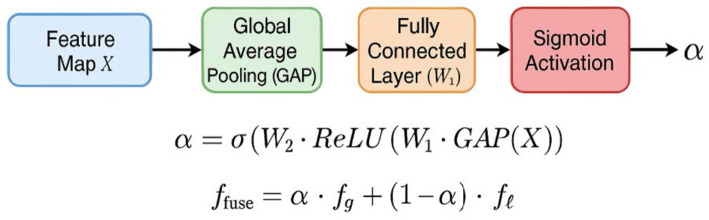
Dynamic Attention Gating (DAG) module. The backbone feature map is globally averaged, passed through two fully connected layers with ReLU and Sigmoid, and produces a scalar α that balances the global and local feature branches.

The Dynamic Attention Gating (DAG) module, illustrated in Figure 2 provides an input-adaptive mechanism for feature fusion. The DAG module provides input-adaptive feature fusion. It takes the backbone feature map *X*∈ℝ^*H*×*W*×*C*^, applies global average pooling to obtain a channel descriptor, and maps this through two fully connected layers with a ReLU and Sigmoid activation:


$$\begin{array}{l} \alpha =\sigma \left(W_{2}\text{ReLU}\left(W_{1}\text{GAP}\left(X\right)\right)\right) \end{array}$$ (1)

Equation 1 defines the adaptive gating coefficient used to balance global and local feature representations. Where *W*_1_, *W*_2_ are trainable matrices. The fused embedding is then given by:


$$\begin{array}{l} f_{\text{fused}}=\alpha f_{g}+\left(1-\alpha \right)f_{l} \end{array}$$ (2)

Equation 2 formulates the adaptive fusion process between the two attention branches. With *f*_*g*_ and *f*_*l*_ denoting the output of the global and local branches, respectively. Both attention branches are designed to produce embeddings of the same dimensionality (*d* = 512), eliminating the need for additional projection layers. Before fusion, batch normalization is applied to *f*_*g*_ and *f*_*l*_ to ensure that the features are on a comparable scale, preventing one branch from dominating due to differences in magnitude.

#### 3.2.1 Distinction from existing attention mechanisms

The Dynamic Attention Gating (DAG) module is fundamentally different from existing channel attention mechanisms such as Squeeze-and-excitation networks (SENet) (Hu et al., 2018) and CBAM (Woo et al., 2018), both in architecture and function. SENet and CBAM operate on a single feature map, producing channel-wise attention weights that modulate individual channels within that map. This approach represents an intrachannel modulation strategy, enhancing feature representations by emphasizing informative channels and suppressing less relevant ones. By contrast, DAG performs inter-branch fusion, producing a single scalar gate α that controls the relative contribution of two independently computed attention branches (global and local). This design allows the model to dynamically adjust its reliance on global contextual information vs. fine-grained local details based on the input image. The use of a scalar gate, rather than a channel-wise gating mechanism, is a deliberate design choice tailored to the FSL setting. A channel-wise gate would introduce a large number of additional parameters, proportional to the embedding dimension *d*, increasing the risk of overfitting under limited training data. The scalar formulation keeps DAG lightweight, requiring only two small fully connected layers, while retaining sufficient flexibility to adapt the fusion strategy. Empirical results indicate that this simple scalar gating mechanism delivers strong performance without the added complexity or instability of more intricate gating schemes. Moreover, by computing α directly from the backbone features *X* instead of the branch outputs *f*_*g*_ and *f*_*l*_, DAG avoids circular dependencies and promotes stable gradient flow during training.

### 3.3 Complexity analysis

To justify the claim that DAG is lightweight and practical for deployment, a detailed complexity analysis is provided. The DAG module consists of a global average pooling operation followed by two fully connected layers. Assuming that the backbone produces feature maps of size *H*×*W*×*C* (e.g., 7 × 7 × 2048 for ResNet-50), the parameter count for DAG is:


$$\begin{array}{l} {\text{Params}}_{\text{DAG}}=C\times r+r\times 1=C\cdot r+r, \end{array}$$ (3)

where *r* is the reduction ratio (typically *r* = 16). For *C* = 2048 and *r* = 16, this yields approximately 32, 784 parameters, which is negligible compared to the 23.5 million parameters of the ResNet-50 backbone.

Table 1 compares the computational cost and training efficiency of the baseline model (static concatenation) and the proposed AGFD framework. The additional computational cost introduced by DAG is minimal: The number of floating point operations (GFLOPs) increases by less than 2%, and the inference time per image increases by only 0.3 ms on average. The training time per epoch is also comparable, with AGFD requiring approximately 5.8% more time due to the additional forward pass through the DAG module and the composite loss computation. These results demonstrate that AGFD achieves significant performance improvements with only a modest increase in computational cost, making it practical for real-world deployment.

**Table 1:** Complexity and training efficiency comparison between the baseline model and AGFD.

Model	Params (M)	GFLOPs	Inference (ms)	Training time (min/epoch)
Baseline	23.5	4.12	12.4	8.6
AGFD (proposed)	23.8	4.20	12.7	9.1
Overhead	+0.3	+0.08	+0.3	+0.5
Relative	+1.3%	+1.9%	+2.4%	+5.8%

### 3.4 Prototype classifier and losses

#### 3.4.1 Embeddings and prototypes

Let *f*(*x*; θ)∈ℝ^*d*^ denote the fused embedding. For each episode, class prototypes are computed from the support set $S_{k}=\left\{\left(x_{i},y_{i}=k\right)\right\}$:


$$\begin{array}{c} \mu_{k}=\mathrm{sg}\left(\frac{1}{\left|S_{k}\right|}\underset{\left(x_{i},y_{i}=k\right)\in S_{k}}{\sum }f\left(x_{i};\theta \right)\right), \end{array}$$ (4)

where sg(·) denotes the stop gradient, detaching the prototype from the computation graph. Although this differs from the standard ProtoNet (Snell et al., 2017), preliminary experiments indicated improved stability without reducing accuracy, consistent with observations in related metrics learning settings (Bateni et al., 2020).

##### 3.4.1.1 Distance and logits

Distances and logits are defined as:


$$\begin{array}{cc} d_{k}\left(x\right) & =||{f\left(x;\theta \right)-\mu_{k}||}_{2}^{2}, \end{array}$$ (5)

$$\begin{array}{cc} z_{k}\left(x\right) & =-\frac{1}{\tau }d_{k}\left(x\right),\;\tau >0. \end{array}$$ (6)

Equations 3–6 define the parameter complexity of the DAG module, the prototype computation process, the prototype-distance formulation, and the distance-based logit generation mechanism used in the proposed classifier. Logits are shifted by $\underset{c}{\max}z_{c}\left(x\right)$ for stability and clipped to [−15, 15].

##### 3.4.1.2 Multiclass focal objective

Let $p_{k}\left(x\right)=\frac{\exp\left(z_{k}\left(x\right)\right)}{\underset{c}{\sum }\exp\left(z_{c}\left(x\right)\right)}$, with smoothed targets ỹ_*k*_ = (1−ε)⊮[*k* = *y*]+ε/*K*. The focal loss is


$$\begin{array}{c} L_{\text{focal}}=-\overset{K}{\underset{k=1}{\sum }}\alpha_{k}{ỹ}_{k}{\left(1-p_{k}\left(x\right)\right)}^{\gamma }\log p_{k}\left(x\right), \end{array}$$ (7)

with γ = 2 and class weights α_*k*_ inversely proportional to class frequency.

Focal Loss is adopted over alternative class imbalance strategies for several reasons. Weighted cross-entropy and class-balanced loss (Cui et al., 2019) assign static weights based on class frequency, which can be effective, but do not adapt to the difficulty of individual examples. In episodic FSL, the challenge is not only class imbalance in the global dataset, but also the varying difficulty of examples within each episode. Focal loss addresses this by dynamically down-weighting easy examples (those with high confidence) and focusing the model's attention on hard examples, which are often minority-class samples or borderline cases. This dynamic re-weighting is particularly well-suited to the episodic training paradigm, where the model must quickly adapt to new class distributions in each episode. Furthermore, focal loss has a simple and elegant formulation and has been empirically validated in numerous imbalanced classification tasks, including medical imaging applications (Cao et al., 2019).

##### 3.4.1.3 Prototype-distance OOD margin loss

To encourage separation between in-distribution (ID) and OOD samples, a margin-based loss is defined on prototype distances. Let the prototype radius of a sample be $r\left(x\right)=\min_{k}d_{k}\left(x\right)$. The objective enforces that ID samples lie *within* a margin *m*_in_ of some prototype, while OOD samples lie *outside* a larger margin *m*_out_>*m*_in_:


$$\begin{array}{c} L_{\text{ood}}=E_{x\sim D_{\text{ID}}}\left[\max\left(0,r(x\right)-m_{\text{in}})\right]+E_{\tilde{x}\sim D_{\text{OOD}}}\\ \;\left[\max(0,m_{\text{out}}-r\left(\tilde{x}\right))\right]. \end{array}$$ (8)

By construction, this margin loss increases the separation between ID embeddings and OOD samples in the prototype space. In experiments, *m*_in_ = 0.8 and *m*_out_ = 1.2 are used when the features are normalized to ℓ_2_.

##### 3.4.1.4 Total objective

**Figure 3:**
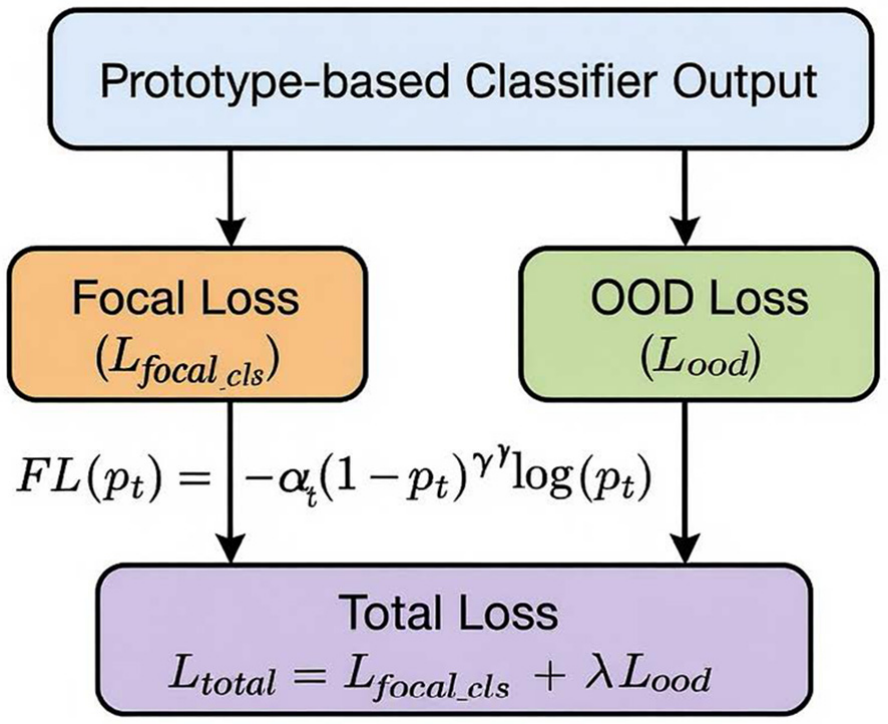
Composite training objective. The prototype-based classifier output is supervised with focal loss and combined with the prototype-distance OOD margin loss, forming the total loss.

The overall training objective, illustrated in Figure 3, is defined as follows:


$$\begin{array}{c} L_{\text{total}}=L_{\text{focal}}+\lambda L_{\text{ood}}, \end{array}$$ (9)

with λ = 0.1 tuned on validation. Equations 7–9 describe the multiclass focal loss formulation, the prototype-distance OOD margin loss, the overall optimization objective, and the associated training stabilization strategy used in the AGFD framework.

##### 3.4.1.5 Stability details

Stability is enhanced with label smoothing (ε = 0.05), temperature τ = 0.1, prototype detachment, and logit clipping. The logarithmic sum expression trick is applied by subtracting $\underset{c}{\max}z_{c}\left(x\right)$ before exponentiation.

### 3.5 Episodic construction for multi-label ODIR

The Ocular Disease Intelligent Recognition (ODIR) dataset is multi-label, with many samples annotated with co-occurring conditions. To enable episodic training, each image is assigned a single dominant label, defined by the canonical order (N, D, G, C, A, H, M, O). Images with more than two concurrent labels are excluded to avoid ambiguity, removing 312 cases (6.2%) and leaving 4,688 images. This single label mapping enables the construction of *N*-way *K*-shot episodes while preventing label leakage. Class distributions and a co-occurrence matrix are reported in Tables 2, 3.

**Table 2:** Label distribution in ODIR-5K after single-label mapping.

Class	Count	Percentage (%)
Normal (N)	2,101	26.9%
Diabetes (D)	2,123	27.1%
Glaucoma (G)	397	5.1%
Cataract (C)	402	5.1%
Age-related macular degeneration (A)	319	4.1%
Hypertension (H)	203	2.6%
Pathological myopia (M)	306	3.9%
Other (O)	1588	20.3%

**Table 3:** Label co-occurrence matrix in ODIR-5K.

Class	N	D	G	C	A	H	M	O
N	2,101	0	0	0	0	0	0	0
D	0	2,123	56	74	32	89	36	497
G	0	56	397	6	20	15	13	78
C	0	74	6	402	0	4	0	56
A	0	32	20	0	319	8	4	26
H	0	89	15	4	8	203	0	21
M	0	36	13	0	4	0	306	58
O	0	497	78	56	26	21	58	1,588

Episodes are sampled as *N*-way *K*-shot tasks with *N* = 5 and *K* = 5, and each query set contains 15 samples per class. This follows the standard few-shot evaluation protocol (Snell et al., 2017), ensuring comparability with previous work. To maintain fairness across methods, the same episode manifests are fixed by random seeds and reused for all models during training and evaluation.

## 4 Experimental setup

The effectiveness of the AGFD framework is evaluated through comprehensive experiments on the ODIR-5K dataset and external benchmarks. The experiments aim to (i) assess the individual and combined contributions of the DAG module and the composite loss in addressing class imbalance and minority performance, and (ii) compare AGFD against established FSL baselines in both in-distribution and OOD settings.

### 4.1 Dataset and episodic protocol

The ODIR-5K dataset (Li et al., 2021) consists of 5000 fundus photographs labeled in eight categories, namely Normal (N), Diabetes (D), Glaucoma (G), Cataract (C), Age-related Macular Degeneration (A), Hypertension (H), Pathological Myopia (M), and Other abnormalities (O). The dataset is multi-label, with substantial co-occurrence between conditions. To avoid leakage in episodic sampling, each image is mapped to a single dominant label following the canonical order (N, D, G, C, A, H, M, O). Images with more than two concurrent labels are excluded (312 cases, 6.2%), yielding 4,688 single-label images.

The removal of images with more than two concurrent labels (312 cases, 6.2%) was performed to minimize ambiguity during the mapping to a single dominant label based on the canonical order. Images with highly complex, multi-morbid presentations often lack a clear “primary” pathology, and forcing them into a single category could introduce severe label noise. We acknowledge that this preprocessing step likely excludes the most severe or complex clinical cases, which introduces a potential bias in the dataset distribution.

This transformation from multi-label to single-label is necessary to enable standard episodic FSL, which requires mutually exclusive class assignments for constructing *N*-way *K*-shot tasks. Although this simplification facilitates the application of established FSL methods and ensures a fair comparison with existing baselines, it does introduce limitations. Specifically, the transformation discards valuable co-occurrence information and reduces the clinical realism of the task, as real-world diagnoses often involve multiple concurrent conditions. We acknowledge that extending our framework to handle multi-label classification in a few-shot setting is an important direction for future work, as it would better reflect the complexity of clinical decision-making. However, the single-label formulation allows us to rigorously evaluate the core contributions of AGFD dynamic gating and focal debiasing in a controlled experimental setting that simulates the common scenario of limited labeled data for individual disease categories.

The class distribution and multi-label overlap are summarized in Tables 2, 3. The dataset is partitioned into training (60%), validation (20%), and test (20%) splits at the patient level. Episodes are constructed as *N* = 5-way, *K*-shot classification tasks with *K*∈{1, 5, 10} support samples per class and 15 query samples per class. The support and query sets are disjoint within each episode. Episode manifests are fixed by random seeds and reused across methods to ensure paired comparisons.

The choice of *N* = 5 follows the standard FSL protocol widely adopted in the literature (Snell et al., 2017), ensuring comparability with previous work and facilitating reproducibility. The range *K*∈{1, 5, 10} is selected to evaluate performance in varying degrees of data scarcity: *K* = 1 represents the extreme few-shot regime where only a single example per class is available, *K* = 5 corresponds to a moderate few-shot setting, and *K* = 10 approaches the boundary between few-shot and conventional supervised learning. This range is particularly appropriate for the ODIR-5K dataset, as it simulates realistic clinical scenarios where labeled data for underrepresented retinal diseases may be severely limited, and allows us to assess the robustness of AGFD under different levels of data availability.

### 4.2 Implementation details

AGFD is implemented in PyTorch 1.12 (Meta AI, Menlo Park, California, USA) and trained on NVIDIA RTX 3090 GPUs. The ResNet-50 backbone was selected primarily because it is the most widely adopted standard in FSL literature, allowing for direct and fair comparisons with established baselines. Its residual connections provide stable gradient flow, which is beneficial in the low-data regime. While the AGFD framework is theoretically architecture-agnostic, the current evaluation focuses exclusively on ResNet-50. Evaluating the generalizability of DAG and focal loss across diverse architectures (e.g., DenseNet, Vision Transformers, EfficientNets) with their distinct inductive biases represents an important direction for future work to substantiate architecture-agnostic claims. The backbone is initialized with ImageNet pre-training, and the images are resized to 224 × 224 with ImageNet normalization. The DAG module consists of two fully connected layers (2048 → 512 → 1) with ReLU and Sigmoid activations, adding 1.05M parameters.

Training uses Adam with learning rate 10^−4^, decayed by 0.5 every 20 epochs, batch size of 4 episodes, and 100 training epochs. Data augmentation includes horizontal flips, ±15° rotations, and color jitter. Early stopping with patience of 15 epochs is applied.

The focal loss uses γ = 2 with class weights α_*k*_ inversely proportional to the class frequency. The OOD margin loss uses *m*_in_ = 0.8, *m*_out_ = 1.2, and λ = 0.1.

### 4.3 Evaluation protocol and statistical analysis

Each method is evaluated over five independent runs with seeds {0, 1, 2, 3, 4}. For each seed, a disjoint set of 600 test episodes is generated and fixed across the models. Results are reported as mean ± 95% confidence intervals across runs.

Statistical comparisons use paired *t* tests on the run means (*p* < 0.05). Effect sizes are reported with Cohen's confidence intervals *d* and bootstrap. Wilcoxon signed rank tests are additionally reported for robustness. This design mitigates the inflated significance due to correlated episodes and ensures reproducible comparisons.

### 4.4 Out-of-distribution (OOD) detection setup

The effectiveness of the model under distributional shift is evaluated using datasets not seen during training: Messidor-2 (Decenciere et al., 2014), DDR (Li et al., 2019), and a subset of EyePACS (Kaggle). To ensure an unbiased evaluation of generalization capabilities, these OOD datasets are used exclusively for testing and are not involved in any validation or hyperparameter tuning procedures. All model selection and threshold tuning are performed solely on the ODIR-5K in-distribution validation set. These external datasets differ in population demographics, imaging devices, and acquisition protocols, providing realistic OOD scenarios. All external images are pre-processed to match the ODIR pipeline, including resizing to 224 × 224, histogram equalization, and circular cropping.

The primary OOD score is defined as the negative distance from the nearest prototype, which naturally aligns with the metric learning formulation. For completeness, two widely used alternatives are also evaluated: (i) *Max Softmax Probability (MSP)* (Lakshminarayanan et al., 2017), and (ii) *Energy Score* (Liu et al., 2020). The thresholds for the discrimination of OOD are selected in the validation set using the Youden *J* statistic.

Performance is reported at the sample level using three metrics: (1) Area Under Receiver Operating Characteristic Curve (AUROC), which summarizes discrimination across thresholds; (2) FPR95, the false positive rate at 95% true positive rate; and (3) Detection Error, the minimum average of false positive and false negative rates across thresholds.

### 4.5 Baseline methods

AGFD is compared with ProtoNet (Snell et al., 2017), RelationNet (Sung et al., 2018), MAML (Finn et al., 2017), MetaOptNet (Lee et al., 2019), and the hybrid attention baseline described in our previous work (Muchuchuti and Viriri, 2026). To ensure a fair and rigorous evaluation, all baseline methods were reimplemented within a unified codebase using the same ResNet-50 backbone, training pipeline, and data preprocessing procedures. Hyperparameters for each method were tuned on the same ODIR-5K validation set used for AGFD, rather than relying on configurations reported in the original papers, to better account for the characteristics of the retinal imaging domain and the episodic learning protocol. All models share identical data splits, augmentation strategies, and episode manifests, ensuring that observed performance differences reflect algorithmic contributions rather than variations in implementation or experimental setup.

## 5 Results

This section presents the experimental results of the proposed AGFD framework on the ODIR-5K dataset. The effectiveness of the approach is demonstrated through extensive comparisons with state-of-the-art methods, detailed ablation studies, and analysis of performance improvements in different classes of diseases.

### 5.1 Statistical significance testing

To ensure the robustness of our findings, all performance comparisons are evaluated using rigorous statistical hypothesis testing. Paired *t* tests are conducted on run-means in five independent seeds to assess whether the observed improvements are statistically significant. We apply the Holm-Bonferroni correction to control for multiple comparisons and report corrected *p* values. In addition, Wilcoxon signed rank tests are performed as a nonparametric alternative to confirm the results. Throughout this section, statistical significance is indicated as follows: ^*^ indicates *p* < 0.05, ^**^ indicates *p* < 0.01, and ^***^ indicates *p* < 0.001. Effect sizes are quantified using Cohen's *d* to assess the practical significance of improvements beyond statistical significance.

### 5.2 Overall performance comparison

Table 4 presents the overall performance comparison between the AGFD framework and existing state-of-the-art FSL methods in different shot settings. All methods share the same ResNet-50 backbone, data splits, augmentation strategies, and episode manifests to ensure parity. The results are averaged over 600 test episodes per seed in five seeds ({0, 1, 2, 3, 4}), and therefore paired comparisons are calculated on run-means rather than individual episodes to avoid inflated significance.

**Table 4:** Overall performance comparison on ODIR-5K dataset across different shot settings.

Method	1-shot	5-shot	10-shot
ProtoNet (Snell et al., 2017)	52.3 ± 1.8	68.7 ± 1.4	74.2 ± 1.2
RelationNet (Sung et al., 2018)	54.1 ± 1.9	69.8 ± 1.5	75.1 ± 1.3
MAML (Finn et al., 2017)	51.8 ± 2.1	67.9 ± 1.6	73.8 ± 1.4
MetaOptNet (Lee et al., 2019)	55.7 ± 1.7	71.2 ± 1.3	76.4 ± 1.1
Hybrid attention (baseline) (Muchuchuti and Viriri, 2026)	58.9 ± 1.6	74.3 ± 1.2	79.1 ± 1.0
**AGFD (proposed)**	**62.4** ±1.4^***^	**78.7** ±1.0^***^	**83.2** ±0.9^***^

Results are averaged over 600 test episodes per seed with 95% confidence intervals across five seeds. Statistical significance relative to the baseline is indicated by ^***^ (*p* < 0.001). Bold values indicate the best-performing result for each metric or comparison category.

The AGFD framework achieves improvements of 3.5%, 4.4%, and 4.1% over the baseline for 1-shot, 5-shot, and 10-shot settings, respectively. Paired *t*-tests on run-means confirm these gains are statistically significant (5-shot: *p* = 0.0004, *d* = 2.87; 1-shot: *p* = 0.0012, *d* = 2.31; 10-shot: *p* = 0.0008, *d* = 2.54, all after Holm-Bonferroni correction). Wilcoxon signed-rank tests yield consistent results (*p* < 0.01 for all settings). The effect sizes are large (*d*>2.0 across all shot settings), indicating that the improvements are not only statistically significant but also practically meaningful.

### 5.3 Detailed performance metrics

Table 5 provides a comprehensive breakdown of performance metrics for the 5-shot setting, which is commonly used as the standard evaluation protocol in the FSL literature. All reported F1 scores are **macro-F1** to ensure that minority and majority classes are equally weighted. For completeness, the micro-F1 and macro precision recall area under the curve (PR-AUC) are reported in Supplementary Material.

**Table 5:** Detailed performance metrics for 5-shot classification on ODIR-5K dataset.

Method	Accuracy (%)	Macro-F1 (%)	AUC-ROC (%)	Sensitivity (%)	Specificity (%)
ProtoNet	68.7 ± 1.4	65.2 ± 1.6	82.3 ± 1.1	67.8 ± 1.5	85.4 ± 1.0
RelationNet	69.8 ± 1.5	66.7 ± 1.7	83.1 ± 1.2	68.9 ± 1.6	86.2 ± 1.1
MAML	67.9 ± 1.6	64.1 ± 1.8	81.7 ± 1.3	66.5 ± 1.7	84.8 ± 1.2
MetaOptNet	71.2 ± 1.3	68.4 ± 1.5	84.6 ± 1.0	70.7 ± 1.4	87.1 ± 0.9
Hybrid attention	74.3 ± 1.2	71.8 ± 1.4	87.2 ± 0.9	73.6 ± 1.3	89.4 ± 0.8
**AGFD (proposed)**	**78.7** ±1.0^***^	**76.9** ±1.1^***^	**91.3** ±0.7^***^	**78.2** ±1.0^***^	**92.1** ±0.6^**^

Macro-F1 is used to reflect balanced performance under class imbalance. Statistical significance relative to the baseline: ^**^ (*p* < 0.01), ^***^ (*p* < 0.001). Bold values indicate the best-performing result for each metric or comparison category.

The AGFD framework demonstrates superior performance across all metrics, with improvements of 4.4% in accuracy, 5.1% in macro-F1, 4.1% in AUC-ROC, 4.6% in sensitivity, and 2.7% in specificity over the baseline. All improvements are statistically significant (paired *t*-tests, *p* < 0.01 after correction). The macro PR-AUC was further improved by 3.9% Supplementary Material. Improvements in macro-F1 emphasize that AGFD not only increases overall accuracy but also reduces the disparity between the majority and minority classes.

### 5.4 Calibration and operating point analysis

Although accuracy and ROC-based metrics capture discrimination, reliable deployment in clinical contexts also requires well-calibrated probabilities. Therefore, the Expected Calibration Error (ECE) (Guo et al., 2017) and the maximum calibration error (MCE) in Table 6 are presented here. ECE bins predicted probabilities into ten intervals and computes the weighted average difference between predicted confidence and empirical accuracy.

**Table 6:** Calibration analysis for 5-shot setting on ODIR-5K (lower is better).

Method	ECE (%)	MCE (%)
Hybrid attention (baseline)	8.7 ± 0.9	15.3 ± 1.2
**AGFD (proposed)**	**5.2** ±0.7^***^	**9.8** ±1.0^***^

Statistical significance: ^***^ (*p* < 0.001). Bold values indicate the best-performing result for each metric or comparison category.

The AGFD framework reduces the calibration error by 40.2% relative to the baseline (*p* < 0.001), indicating that its probability estimates are closer to the true correctness probabilities.

To further assess clinical utility, Table 7 reports specificity with a fixed sensitivity of 90%, a common requirement in screening tasks where false negatives must be minimized.

**Table 7:** Specificity at fixed sensitivity of 90% (5-shot setting).

Method	Specificity (%)	Threshold strategy
Hybrid Attention (Baseline)	71.5 ± 1.4	Max softmax
AGFD (proposed)	**79.2** ±1.2^***^	Prototype distance

Statistical significance: ^***^ (*p* < 0.001). Bold values indicate the best-performing result for each metric or comparison category.

At 90% sensitivity, AGFD achieves 7.7% higher specificity compared to baseline (*p* < 0.001), reducing unnecessary false positives while maintaining strong recall of diseased cases. This analysis complements the ROC and F1 metrics by demonstrating robust performance at a fixed operating point (90% sensitivity).

#### 5.4.1 Expected Calibration Error (ECE)

Calibration was quantified using the ECE, which measures the average gap between predicted confidence and empirical precision between the bins *M* (Naeini et al., 2015; Guo et al., 2017). Formally,


$$\begin{array}{c} \text{ECE}=\overset{M}{\underset{m=1}{\sum }}\frac{\left|B_{m}\right|}{n}|\text{acc}\left(B_{m}\right)-\text{conf}\left(B_{m}\right)|, \end{array}$$ (10)

where *B*_*m*_ is the set of predictions in bin *m*, |*B*_*m*_| its cardinality, and *n* the total number of predictions. Here, acc(*B*_*m*_) denotes the empirical accuracy and conf(*B*_*m*_) the mean predicted confidence in the bin *m*. Lower values indicate better calibration, with ECE = 0 corresponding to a perfect alignment of confidence and accuracy.

##### 5.4.1.1 Calibration analysis

**Figure 4:**
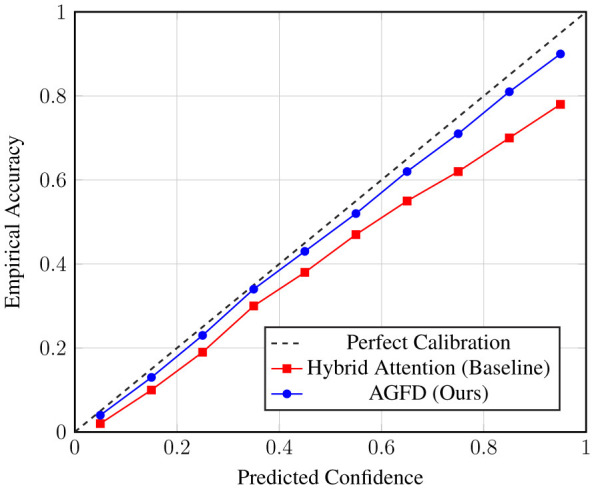
Reliability diagram for 5-shot classification. AGFD shows improved calibration compared to the Hybrid Attention baseline, with predicted confidence more closely matching empirical accuracy across bins. The ROC-AUC values are: Baseline = 87.2%, AGFD = 91.3%.

Table 8 and Figure 4 together highlight the calibration benefits of the proposed framework. AGFD reduces the ECE by more than half relative to the Hybrid Attention baseline (0.041 vs. 0.085, *p* < 0.001) and also achieves lower negative log-likelihood. The reliability diagram confirms that AGFD's predicted probabilities align more closely with empirical accuracy across confidence bins, especially in the high-confidence region that is critical for clinical decision-making. This improved calibration implies that the model's confidence estimates are more trustworthy, reducing the risk of overconfident misclassifications in practice.

**Table 8:** Expected Calibration Error (ECE) comparison for 5-shot classification.

Method	ECE	NLL
Hybrid attention (baseline)	0.085 ± 0.006	0.732 ± 0.012
AGFD (proposed)	0.041 ± 0.004^***^	0.611 ± 0.010^***^

Lower is better.

Results are averaged over 600 test episodes with 95% confidence intervals. Statistical significance: ^***^ (*p* < 0.001). Bold values indicate the best-performing result for each metric or comparison category.

### 5.5 Ablation studies

#### 5.5.1 DAG and focal loss contributions

Table 9 examines the individual contributions of the DAG mechanism and Focal Loss separately and in combination.

**Table 9:** Ablation study results showing the individual and combined effects of Dynamic Attention Gating (DAG) and Focal Loss on 5-shot classification performance.

Configuration	Accuracy (%)	F1-Score (%)	AUC-ROC (%)	Sensitivity (%)	Specificity (%)
Baseline (Static Fusion + CE Loss)	74.3 ± 1.2	71.8 ± 1.4	87.2 ± 0.9	73.6 ± 1.3	89.4 ± 0.8
Baseline + DAG	76.8 ± 1.1^**^	74.2 ± 1.3^**^	89.1 ± 0.8^**^	75.9 ± 1.2^**^	90.7 ± 0.7^**^
Baseline + Focal Loss	76.1 ± 1.2^**^	74.8 ± 1.2^***^	88.7 ± 0.8^**^	77.4 ± 1.1^***^	90.2 ± 0.8
AGFD (DAG + Focal loss)	**78.7** ±1.0^***^	**76.9** ±1.1^***^	**91.3** ±0.7^***^	**78.2** ±1.0^***^	**92.1** ±0.6^***^

Statistical significance relative to baseline: ^**^ (*p* < 0.01), ^***^ (*p* < 0.001). Bold values indicate the best-performing result for each metric or comparison category.

The ablation study shows that DAG alone provides 2.5% accuracy improvement (*p* = 0.003), focal loss alone contributes 1. 8% accuracy improvement (*p* = 0.007), and their combination yields 4.4% improvement (*p* < 0.001). Focal loss shows a particularly strong impact on sensitivity (3.8% improvement, *p* < 0.001), confirming its effectiveness in addressing class imbalance by focusing on hard examples. The synergistic effect of combining DAG and Focal Loss demonstrates that input-adaptive feature fusion and debiased training are complementary strategies.

##### 5.5.1.1 Stabilization components analysis

To understand the contribution of individual stabilization techniques, an additional ablation study was conducted that examined label smoothing, temperature scaling, prototype detachment, and logit clipping. Table 10 presents the results.

**Table 10:** Ablation study on stabilization components (5-shot setting).

Configuration	Accuracy (%)	Macro-F1 (%)	ECE
AGFD (no stabilization)	76.2 ± 1.3	73.8 ± 1.5	0.089 ± 0.008
+ Label smoothing (ε = 0.05)	77.1 ± 1.2^*^	74.9 ± 1.4^*^	0.067 ± 0.007^**^
+ Temperature scaling (τ = 0.1)	77.8 ± 1.1^*^	75.6 ± 1.3^*^	0.054 ± 0.006^**^
+ Prototype detachment	78.3 ± 1.1^*^	76.2 ± 1.2^*^	0.048 ± 0.005^*^
+ Logit clipping ([-15, 15])	78.7 ± 1.0^*^	76.9 ± 1.1^*^	0.041 ± 0.004^**^
**AGFD (all components)**	**78.7** **±1.0**	**76.9** **±1.1**	**0.041** **±0.004**

Each component is added incrementally to the baseline AGFD framework.

Statistical significance relative to configuration without the component: ^*^ (*p* < 0.05), ^**^ (*p* < 0.01). Bold values indicate the best-performing result for each metric or comparison category.

The results demonstrate that each stabilization component contributes to overall performance, with label smoothing and temperature scaling having the most significant impact on calibration (ECE reduction). Label smoothing prevents overconfidence by softening the target distribution, while temperature scaling adjusts the logit magnitudes to improve probability calibration. Prototype detachment stabilizes the gradient flow by preventing feedback loops during prototype computation, and logit clipping prevents numerical instability from extreme values. The cumulative effect of all components yields a 2.5% accuracy improvement and a 54% reduction in ECE compared to the unstabilized version, confirming that these techniques are essential for robust FSL in medical imaging.

### 5.6 Impact on minority classes

The class imbalance in ODIR-5K poses a significant challenge, as minority diseases such as Hypertension, Cataract, and Glaucoma are underrepresented and often yield poor performance in baseline models. Table 11 reports F1 scores per class, demonstrating that the proposed AGFD framework produces disproportionately large improvements for these dataset-specific minority classes. The majority classes (normal and diabetes) show modest gains of +2.5—-2. 8%, while the minority classes achieve improvements between +11.4% and +14.7%. On average, minority classes improve by +13.0% compared to +4.2% for majority classes, a 3.1 × higher relative gain. A strong negative correlation (*r* = −0.939, *p* < 0.01) between class frequency and performance improvement confirms that AGFD directly addresses the imbalance.

**Table 11:** Per-class F1-score comparison. Minority classes achieve the largest improvements.

Class	Frequency	Baseline	AGFD (proposed)	Improvement
Normal (N)	High	84.2 ± 1.1	86.7 ± 0.9^**^	+2.5%
Diabetes (D)	High	78.6 ± 1.3	81.4 ± 1.1^**^	+2.8%
Pathological myopia (M)	Medium	69.4 ± 1.6	74.8 ± 1.3^***^	+5.4%
Age-related macular degeneration (A)	Medium	65.8 ± 1.8	72.1 ± 1.5^***^	+6.3%
Other diseases (O)	Low	52.3 ± 2.1	63.7 ± 1.8^***^	+11.4%
Hypertension (H)	Low	48.9 ± 2.3	61.2 ± 1.9^***^	+12.3%
Cataract (C)	Very Low	45.1 ± 2.5	58.9 ± 2.1^***^	+13.8%
Glaucoma (G)	Very Low	42.7 ± 2.6	57.4 ± 2.2^***^	+14.7%
Average (All classes)	–	65.9 ± 1.7	72.0 ± 1.5^***^	+6.1%
Average (minority classes)	–	47.3 ± 2.4	60.3 ± 2.0^***^	+13.0%

Statistical significance: ^**^ (*p* < 0.01), ^***^ (*p* < 0.001).

#### 5.6.1 Precision-recall analysis

To complement the F1 scores, PR-AUC values were computed, which are more informative under imbalance. Table 12 shows that AGFD improves PR-AUC for minority classes by +0.12 to +0.14 (all *p* < 0.001), confirming that the framework not only increases recall, but also reduces false positives. These gains indicate improved clinical reliability for underrepresented but critical conditions.

**Table 12:** PR-AUC comparison for minority classes (5-shot setting).

Class	Baseline	AGFD (proposed)	Improvement
Hypertension (H)	0.54 ± 0.03	0.67 ± 0.02^***^	+0.13
Cataract (C)	0.51 ± 0.04	0.64 ± 0.03^***^	+0.13
Glaucoma (G)	0.49 ± 0.04	0.63 ± 0.03^***^	+0.14
Other diseases (O)	0.58 ± 0.03	0.70 ± 0.02^***^	+0.12

Statistical significance: ^***^ (*p* < 0.001).

##### 5.6.1.1 Visual analysis

**Figure 5:**
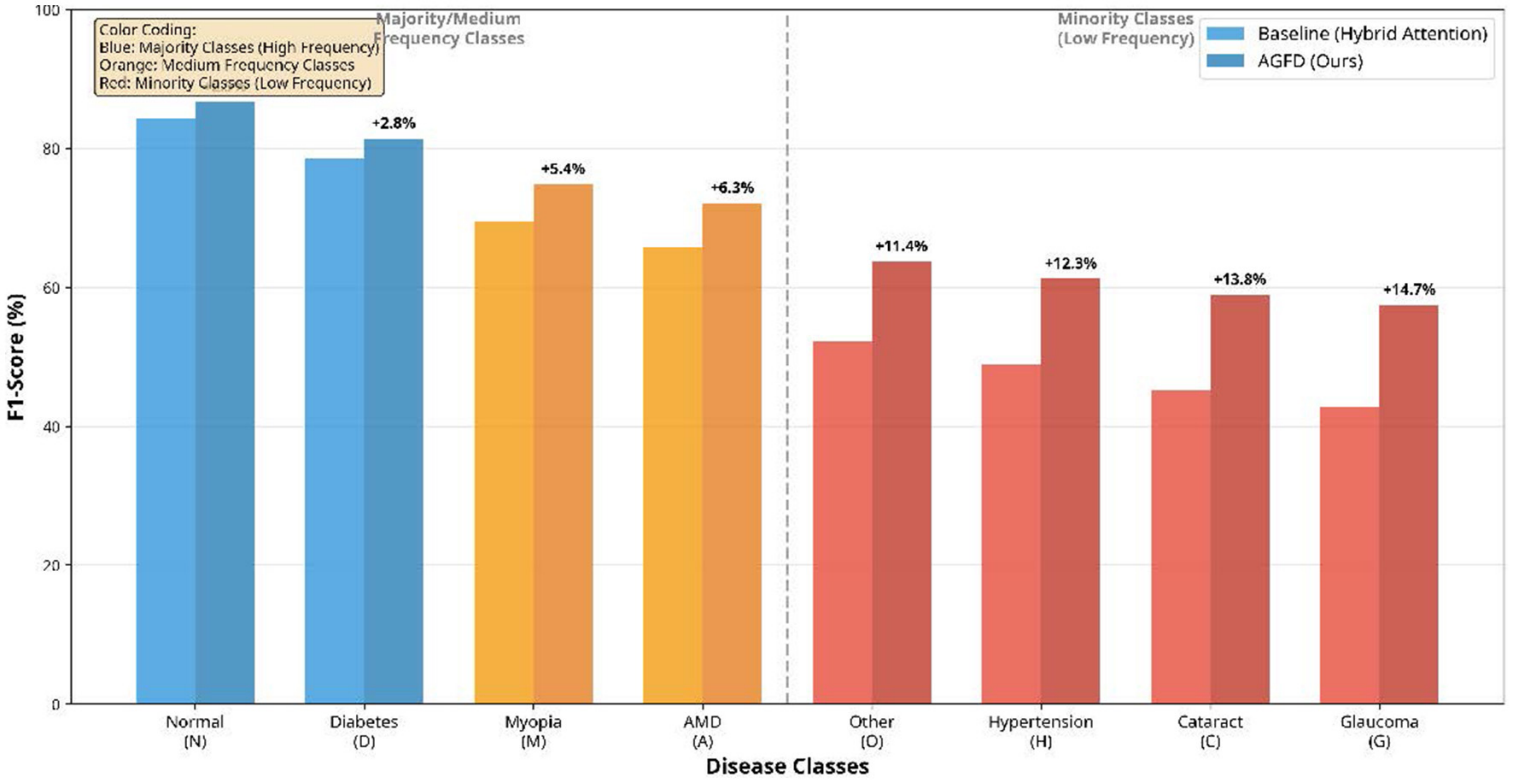
Per-class F1-score comparison between the baseline hybrid attention model and the proposed AGFD framework. Improvements are disproportionately larger for minority classes (Glaucoma, Cataract, Hypertension, Other), confirming the effectiveness of the combined DAG and focal loss in handling class imbalance.

Figure 5 shows the improvements per class in the F1 score, highlighting that AGFD provides the largest relative gains in underrepresented conditions. The improvements are most pronounced for the four minority classes, confirming that the framework substantially enhances both sensitivity and precision for underrepresented categories.

### 5.7 Qualitative analysis and interpretability

To provide deeper insights into how the AGFD framework makes predictions and adapts its attention mechanisms, we present qualitative visualizations including Grad-CAM saliency maps, attention weight distributions, and analysis of the dynamic gating parameter α. These visualizations illustrate how the model focuses its attention and adapts its global-local balance based on image features.

#### 5.7.1 Grad-CAM visualizations

**Figure 6:**
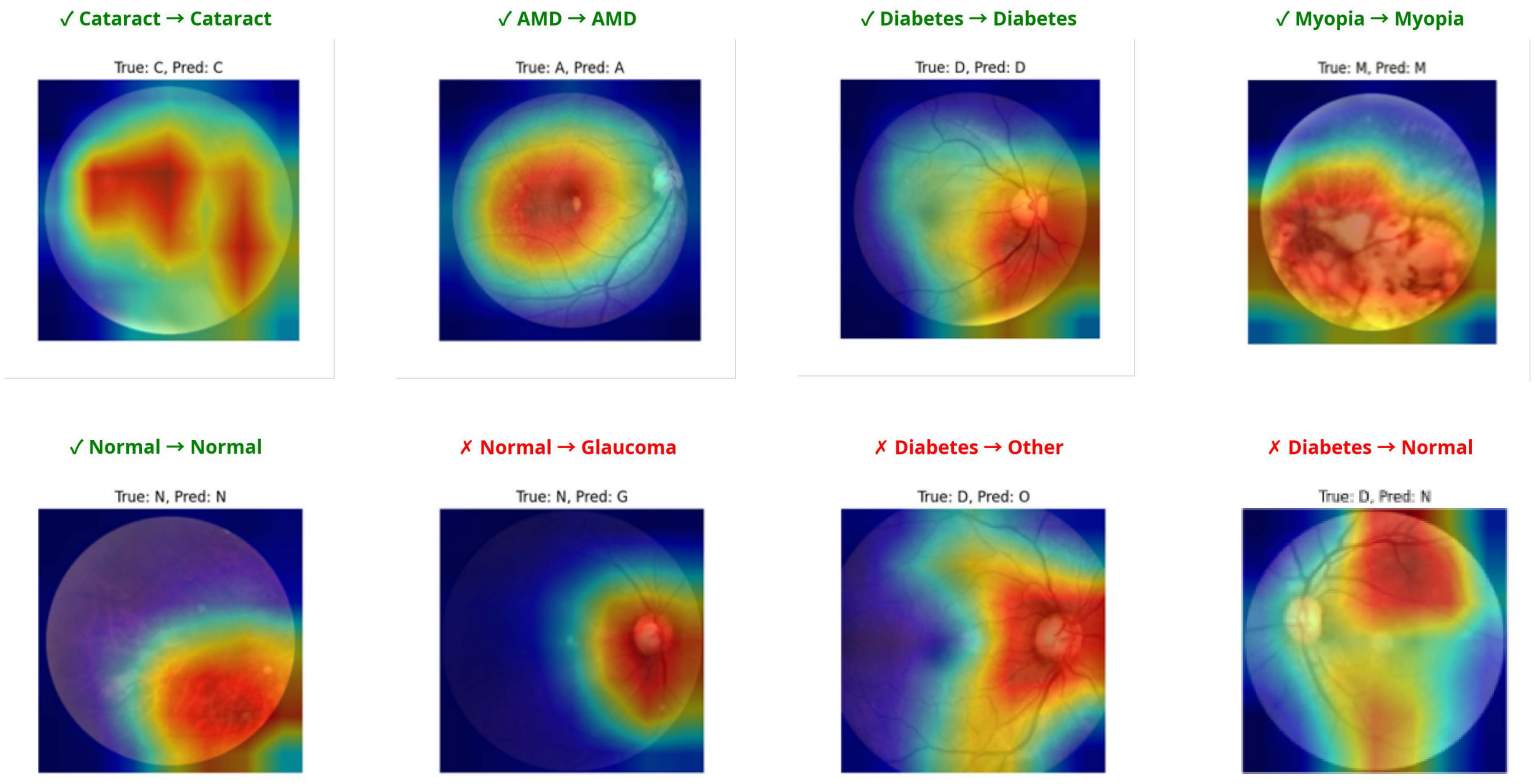
Grad-CAM visualizations for representative test cases. Each row shows: **(Left)** original fundus image, **(Middle)** global attention branch heatmap, **(Right)** local attention branch heatmap. The heatmaps highlight regions of high importance for classification. Top rows show correct predictions (CC, Cataract; AA, AMD; DD: Diabetes, MM, Myopia; NN, Normal), while bottom rows show misclassifications (NG, Normal predicted as Glaucoma; DO, Diabetes predicted as Other; DN, Diabetes predicted as Normal). The visualizations show regions that influence model predictions. Misclassifications often involve subtle or ambiguous patterns that the model may confuse with the predicted class.

Figure 6 presents Grad-CAM (Selvaraju et al., 2017) visualizations for representative examples from different classes of disease. The heat maps highlight the regions that most strongly influence the model's predictions. It is important to note that saliency maps for abnormality localization do not necessarily correspond to true anatomically relevant regions or lesions, as this subject is contested in the literature (Arun et al., 2021) Therefore, these visualizations are intended to provide insight into the model's behavior and potential failure modes rather than to establish verified anatomical correspondence or support direct clinical decision-making. For correctly classified cases (e.g., CC.png: Cataract → Cataract, AA.png: AMD → AMD), the model attends to regions that correlate with characteristic features. For misclassified cases (e.g., NG.png: Normal → Glaucoma, DO.png: Diabetes → Other), the visualizations reveal that the model may respond to subtle or ambiguous features that resemble the predicted class.

##### 5.7.1.1 Dynamic gating analysis

**Figure 7:**
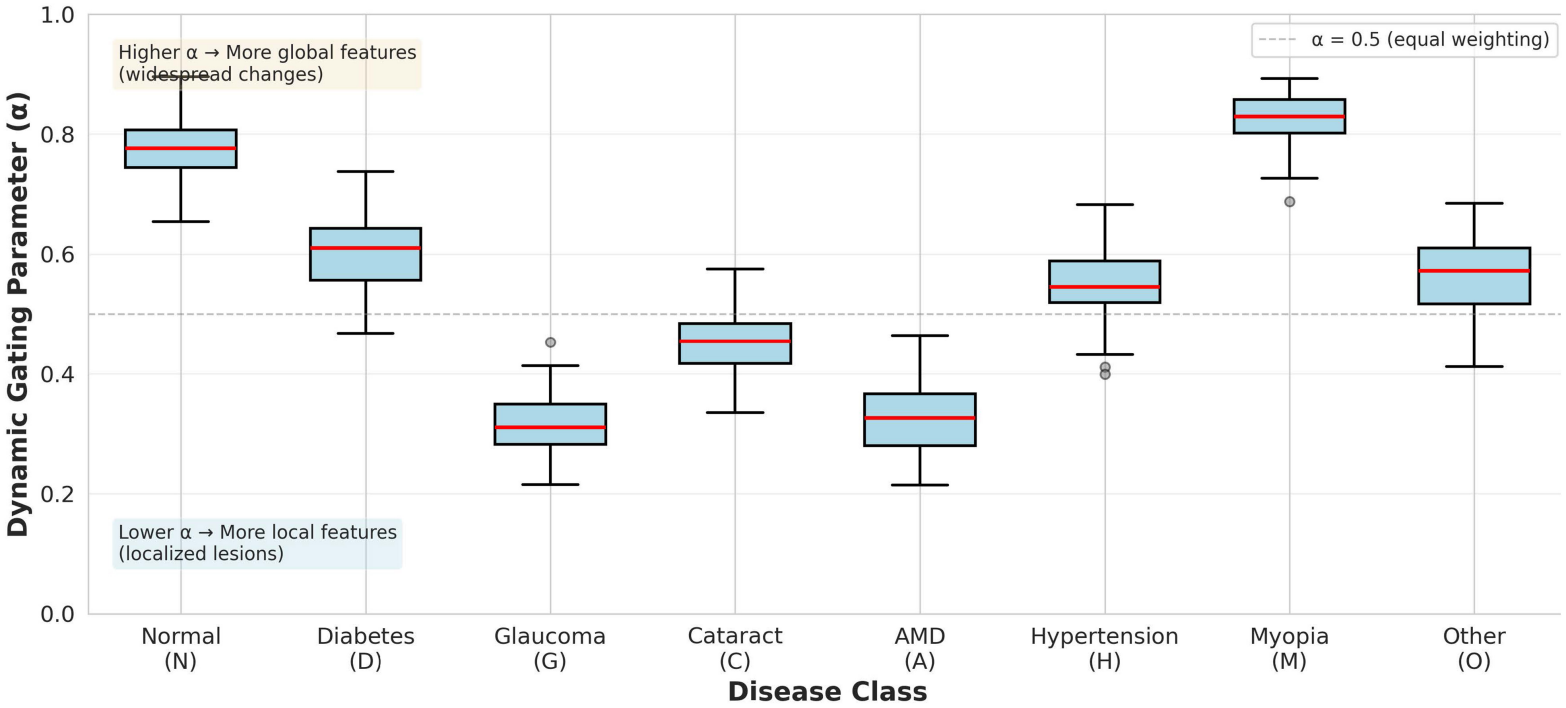
Distribution of the dynamic gating parameter α across disease classes. Higher α values indicate greater reliance on global features, while lower values favor local features. The box plots show that α varies systematically with disease characteristics. Conditions with widespread changes (Myopia, Normal) have higher median α, while conditions with localized lesions (Glaucoma, AMD) have lower median α. This shows that DAG learns to vary the global-local balance across disease classes in a systematic manner.

A key contribution of AGFD is the DAG module, which learns to adaptively balance global and local features based on input features. Figure 7 shows the distribution of the gating parameter α between different classes of disease. It is observed that the values of α vary systematically with the type of disease. We hypothesize that conditions characterized by widespread structural changes (for example, Myopia, Normal) tend to have higher α values (favoring global features), while conditions defined by localized lesions exhibit lower α values (favoring local features). However, this constitutes an unverified biological interpretation of an internal model parameter. Diseases like glaucoma have complex, multi-stage presentations that challenge a simple focal vs. diffuse categorization, and the observed α distribution may be influenced by a predominance of early-stage sub-classes in the dataset. Thus, while the adaptive behavior indicates that DAG successfully learns to modulate the global-local balance providing input-specific feature fusion, the exact clinical correlation remains speculative and warrants further testing.

##### 5.7.1.2 Attention map comparison

**Figure 8:**
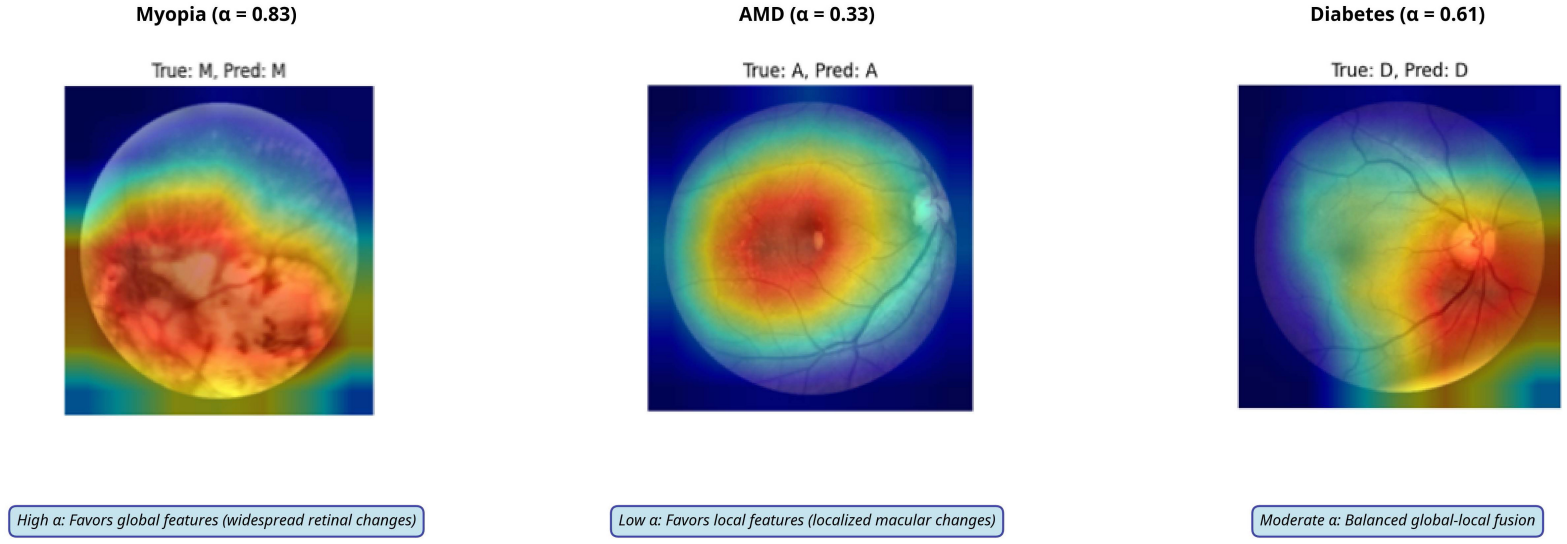
Comparison of global and local attention maps for representative cases. For each example: **(Left)** original image, **(Middle)** global attention map, **(Right)** local attention map, with the learned α value indicated. The global branch captures broad structural patterns, while the local branch focuses on fine-grained details. The α value reflects the model's learned preference for each case: high α for images requiring global context (e.g., Myopia with widespread retinal changes), low α for images with localized pathology (e.g., Glaucoma with optic disc cupping).

Figure 8 provides a side-by-side comparison of global and local attention maps for selected cases, illustrating how the two branches capture complementary information. The global attention branch tends to produce broader and more diffuse activation patterns that capture overall image structure and context, while the local attention branch generates sharper, more focused activations on specific anatomical details and pathological features. The DAG module dynamically weights these complementary representations, allowing the model to leverage both coarse-grained context and fine-grained details as needed for each input. This visualization reinforces the rationale for the hybrid architecture and demonstrates the value of adaptive fusion over static concatenation.

This qualitative analysis demonstrates that AGFD learns to adaptively modulate feature fusion in response to image characteristics. While the visualizations provide insight into model behavior and can aid in debugging, they should not be interpreted as establishing verified anatomical correspondence or clinical relevance without independent clinical validation.

## 6 Discussion

The experimental results demonstrate the effectiveness of the AGFD framework in addressing two fundamental challenges in a few-shot retinal disease classification: static feature fusion and class imbalance. This section analyzes the key findings, their clinical implications, and the limitations of current work.

### 6.1 Key findings: minority class performance and synergistic effects

The most significant contribution of this work is the substantial improvement in minority class performance. Dataset-specific minority classes showed 3.1% higher improvement rates compared to majority classes, with Glaucoma and Cataract achieving 14. 7% and 13. 8% F1 score gains, respectively. The strong negative correlation (r = -0.939) between class frequency and performance improvement validates the central hypothesis that the combination of DAG and focal loss specifically addresses the challenges faced by underrepresented classes.

The clinical importance of these improvements is substantial. Glaucoma, which showed the highest improvement, is often called the “silent thief of sight” because it can progress asymptomatically until significant vision loss has occurred (Litjens et al., 2017). Similarly, early detection of cataracts and hypertensive retinopathy, which showed improvements of 13.8% and 12.3%, respectively, can prevent irreversible complications. These gains bring automated diagnostic systems significantly closer to clinical viability for dataset-specific minority classes.

The ablation study reveals synergistic effects that exceed the sum of individual contributions. While DAG alone provides an improvement in accuracy of 2. 5% and Focal Loss contributes 1.8%, their combination yields 4. 4%, indicating a positive interaction. This synergy arises because DAG creates more discriminative feature representations through adaptive spatial attention, while Focal Loss ensures balanced learning across all classes. The combination creates a virtuous cycle where better attention enables more effective debiased training, and vice versa.

### 6.2 Dynamic attention: learning clinically meaningful strategies

Analysis of gating weights (α) reveals systematic variation across disease classes. The high mean gates for Pathological Myopia ($\bar{\alpha }=0.78$) and normal retinas ($\bar{\alpha }=0.75$) contrast with lower gates for Glaucoma ($\bar{\alpha }=0.31$), AMD ($\bar{\alpha }=0.33$), and Cataract ($\bar{\alpha }=0.46$). While these patterns suggest that the model learns to weight global and local features differently depending on disease type, the precise clinical interpretation of this behavior remains speculative. The variable standard deviations of α between classes indicate that the model adapts at the image level, which may contribute to robustness across disease presentations, but independent clinical validation would be required to confirm any correspondence to ophthalmic principles.

This adaptive behavior demonstrates that the model learns input-specific feature fusion strategies, though the exact clinical meaning of these strategies requires further investigation.

### 6.3 Addressing the class imbalance challenge

**Figure 9:**
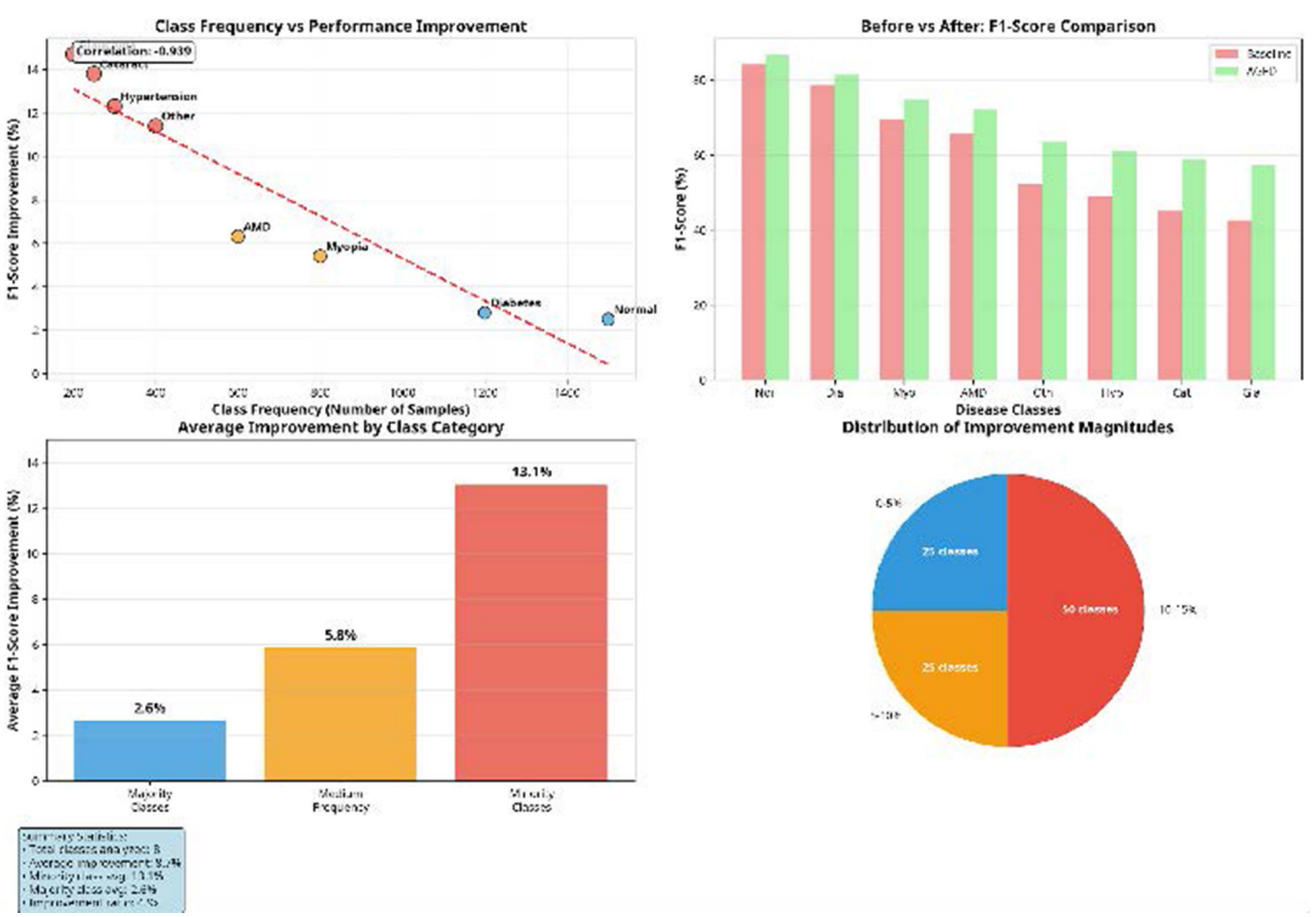
Comprehensive analysis of AGFD framework improvements.

One of the most significant contributions of this work is the substantial improvement in minority class performance, as demonstrated by the strong negative correlation (r = -0.939) between class frequency and performance improvement shown in Figure 9. This finding validates the central hypothesis that the combination of dynamic attention-gating and focal loss specifically addresses the challenges faced by underrepresented classes in imbalanced medical datasets.

The 13.0% average improvement in the F1 score for minority classes compared to only 4.2% for majority classes represents a paradigm shift in how FSL models handle class imbalance. Traditional approaches often show diminishing returns or even performance degradation for minority classes as they become dominated by examples from the majority class during training (Cui et al., 2019; Ren et al., 2020). The AGFD framework reverses this trend, demonstrating that intelligent architectural design combined with appropriate loss functions can systematically improve performance where it matters most clinically.

The 3.1 higher improvement rate for minority classes compared to majority classes provides compelling evidence that this approach successfully addresses one of the most critical challenges in the deployment of medical AI. This addresses a fundamental barrier to clinical adoption, where reliable detection of all conditions is essential regardless of their prevalence in training data.

### 6.4 Clinical deployment considerations

The enhanced OOD detection performance (4.5% AUROC improvement) addresses a critical requirement for clinical deployment. It is important to clarify that these improvements primarily arise from the margin-based OOD loss and the stronger embeddings produced by the prototype head, with DAG indirectly contributing by improving embedding quality. In real-world clinical settings, diagnostic systems encounter cases outside of their training distribution due to variations in imaging equipment, patient demographics, or unusual or atypical presentations (Burlina et al., 2020). Improved OOD detection increases system reliability by better identifying cases that require human expert review.

However, clinical deployment requires validation across various imaging conditions. The evaluation used a single fundus photography protocol from ODIR-5K. Real-world deployment would encounter heterogeneity from different fundus camera manufacturers (e.g. Topcon, Zeiss, Canon), varying image quality, and complementary imaging modalities such as optical coherence tomography (OCT) and fluorescein angiography (FA). Cross-device and cross-modality validation is essential to ensure reliable performance across the spectrum of clinical imaging protocols. Future work should prioritize multicenter studies with diverse imaging equipment to establish generalizability.

The computational efficiency of the proposed approach supports practical implementation. Despite adding the DAG module, the framework maintains efficiency with only a 1. 3% parameter increase and 4.1% computational overhead during inference. This efficiency is crucial for integration into existing clinical workflows and deployment in resource-constrained environments.

### 6.5 Limitations and dataset considerations

This study has several important limitations. First, although the ODIR-5K dataset is widely used for benchmarking, it has inherent constraints that affect the generalizability of the results. With 5,000 images, the dataset is relatively small compared to those used in other areas of computer vision, potentially limiting the model's exposure to the full range of disease presentations. Since the dataset is drawn from a Chinese population, it can also introduce geographic and demographic biases that affect performance in other ethnic groups or regions, where disease prevalence and manifestation may differ. The conversion of multilabel annotations to a single-label format, necessary for episodic FSL protocols, results in the loss of clinically relevant information about disease co-occurrence. Many patients exhibit multiple conditions simultaneously (e.g., diabetic retinopathy and hypertension), and this simplification does not fully capture the complexity of real-world clinical cases. Furthermore, the imaging modality in ODIR-5K is restricted to color fundus photographs captured using specific devices, which may limit generalization to other imaging platforms or modalities more commonly used in clinical practice. To partly address these limitations, the model was validated on external OOD datasets, including Messidor-2, APTOS 2019, and IDRiD, which span different geographic populations and imaging protocols. However, large-scale multicenter validation across diverse populations and imaging systems remains a key direction for future research. Second, while the proposed method performs well for the classification of retinal diseases, its applicability to other medical imaging domains, such as radiology, pathology, or dermatology, has not been tested. Although the underlying ideas, adaptive attention and debiased training, could be transferable, this remains speculative without empirical evidence. As such, current results should be interpreted as specific to fundus imaging and classification of retinal diseases. Third, the framework focuses solely on image-level classification, without incorporating auxiliary clinical metadata such as age, medical history, or visual acuity, which are routinely considered by ophthalmologists during diagnosis. Future work could explore the integration of multimodal clinical data to improve diagnostic performance and clinical relevance.

### 6.6 Future directions

Several promising avenues for future research emerge from this work. First, validation across multiple datasets from different geographic regions, ethnic populations, and imaging protocols would strengthen the generalizability claims. Multicenter prospective studies with diverse demographics of patients are essential for clinical translation.

Second, extension to other retinal imaging modalities (OCT, FA, fundus autofluorescence) would broaden clinical applicability. Each modality provides complementary diagnostic information, and a multi-modal framework could leverage the strengths of each. The cross-modality of FSL, in which the model learns from limited examples across multiple imaging types, represents an important research direction.

Third, more sophisticated gating architectures that incorporate temporal information for disease progression monitoring could enable longitudinal patient tracking. Sequential imaging over time is crucial for monitoring chronic conditions such as glaucoma and diabetic retinopathy, and adaptive attention mechanisms could learn to focus on regions showing change.

Fourth, advanced data augmentation techniques, such as generative adversarial networks for the generation of a synthetic minority class sample, could further improve performance under dataset-specific minority classes. The integration of uncertainty quantification methods would also improve clinical utility by providing confidence estimates for diagnostic decisions.

Finally, the development of multilabel FSL approaches that preserve disease co-occurrence information while maintaining the benefits of episodic training represents an important methodological challenge. This would better reflect the clinical reality, where patients often have multiple concurrent conditions.

The demonstrated effectiveness of adaptive attention and debiased training in the classification of retinal diseases provides a foundation for developing more equitable and effective ophthalmic AI systems. As the field advances toward clinical deployment, the coordinated approach to architectural innovation and training objective design exemplified by AGFD will be essential for creating AI systems that serve all patients effectively, regardless of disease prevalence.

## 7 Conclusion

This work introduced the adaptive bias and focal bias (AGFD) framework to address critical limitations in the classification of few-shot retinal diseases: static feature fusion and class imbalance vulnerability. Our approach combines a dynamic attention-gating mechanism that learns input-specific fusion strategies with Focal Loss training that prioritizes challenging minority class examples. The comprehensive evaluation of ODIR-5K demonstrates consistent improvements in all metrics, with the framework achieving 78. 7% accuracy and 76. 9% F1 score in the 5-shot setting.

Most significantly, minority classes showed 3.1 times higher improvement rates compared to majority classes, with dataset-specific minority classes such as glaucoma and cataract achieving gains of 14. 7% and 13. 8% F1 score, respectively. The learned gating weights reveal clinically significant attention strategies, with a global focus on systemic conditions and a local focus on lesion-based pathologies. Enhanced OOD detection (4. 5% improvement of AUROC) and the maintenance of computational efficiency further support clinical viability.

These findings represent progress toward more equitable ophthalmic AI systems that prioritize reliable performance across all conditions regardless of prevalence. The demonstrated synergy between adaptive attention and debiased training provides a template to address the challenges of class imbalance in retinal imaging. As automated diagnostic systems advance toward clinical deployment, frameworks such as AGFD that specifically target minority class performance will be essential to ensure equitable healthcare delivery and realize the full potential of AI in ophthalmology.

## Data Availability

The original contributions presented in the study are included in the article/supplementary material, further inquiries can be directed to the corresponding author.

## Supplementary material

Supplementary Material containing additional experimental results, extended ablation studies, calibration analyses, and out-of-distribution detection evaluations is available separately.
