# Supplementary material for: Adaptive Gating and Focal Debiasing for robust few-shot retinal disease classification
Source: Front Artif Intell. 2026 May 26;9:1825132. doi: 10.3389/frai.2026.1825132 (PMC13246412; doi:10.3389/frai.2026.1825132)
Supplement: Supplementary file 2 [file Supplementary_file_2.pdf]

Supplementary Material

1 SUPPLEMENTARY RESULTS

This supplementary material provides additional performance metrics, detailed curves, and analysis that complement the main manuscript.

1.1 Micro-F1 and Macro PR-AUC Results

Table S1 presents micro-F1 scores and macro Precision-Recall Area Under the Curve (PR-AUC) values for all methods in different shot settings. Although the main manuscript focuses on macro-F1 to emphasize balanced performance across classes, micro-F1 provides an overall accuracy-weighted perspective. The macro PR-AUC complements the ROC-AUC analysis by focusing on performance in imbalanced scenarios where the positive class is underrepresented.

Table S1. Micro-F1 and Macro PR-AUC scores across different shot settings. Results are averaged over 600 test episodes with 95% confidence intervals.

Method	Micro-F1 (%)			Macro PR-AUC (%)		
	1-shot	5-shot	10-shot	1-shot	5-shot	10-shot
ProtoNet	68.2 ± 1.5	68.7 ± 1.4	69.3 ± 1.3	64.8 ± 1.6	65.4 ± 1.5	66.1 ± 1.4
RelationNet	69.3 ± 1.6	69.8 ± 1.5	70.4 ± 1.4	65.9 ± 1.7	66.5 ± 1.6	67.2 ± 1.5
MatchingNet	67.8 ± 1.7	68.3 ± 1.6	68.9 ± 1.5	64.2 ± 1.8	64.8 ± 1.7	65.5 ± 1.6
MAML	70.1 ± 1.4	70.6 ± 1.3	71.2 ± 1.2	66.7 ± 1.5	67.3 ± 1.4	68.0 ± 1.3
Meta-Baseline	71.5 ± 1.3	72.0 ± 1.2	72.6 ± 1.1	68.1 ± 1.4	68.7 ± 1.3	69.4 ± 1.2
Hybrid Attention (Baseline)	73.8 ± 1.2	74.3 ± 1.1	74.9 ± 1.0	70.4 ± 1.3	71.0 ± 1.2	71.7 ± 1.1
**AGFD (Proposed)**	**77.1** ± **1.0**	**78.7** ± **0.9**	**79.3** ± **0.8**	**74.3** ± **1.1**	**74.9** ± **1.0**	**75.6** ± **0.9**
Improvement over Baseline	+3.3	+4.4	+4.4	+3.9	+3.9	+3.9

The AGFD framework achieves consistent improvements in both micro-F1 and macro PR-AUC in all shot settings. The micro-F1 improvements of 3.3–4.4% demonstrate that AGFD enhances the overall classification accuracy. More importantly, the macro PR-AUC improvements of 3.9% confirm that AGFD significantly improves performance in minority classes, which is critical for clinical deployment in imbalanced medical imaging scenarios.

1.2 ROC and PR Curves

Figures S1, S2, and S3 present a comprehensive analysis of the ROC and PR curves for all classes and macro-averaged performance.

Figure S1: Per-class ROC curves comparing baseline and AGFD for 5-shot classification. All classes show improved AUC with AGFD (dashed lines) compared to baseline (solid lines), with the largest improvements observed for minority classes (Glaucoma: +8.9%, Cataract: +8.9%, Hypertension: +8.7%).

The ROC curves (Figure S1) demonstrate that AGFD improves classification performance in all classes, with particularly strong gains for minority classes. The PR curves (Figure S2) are especially informative

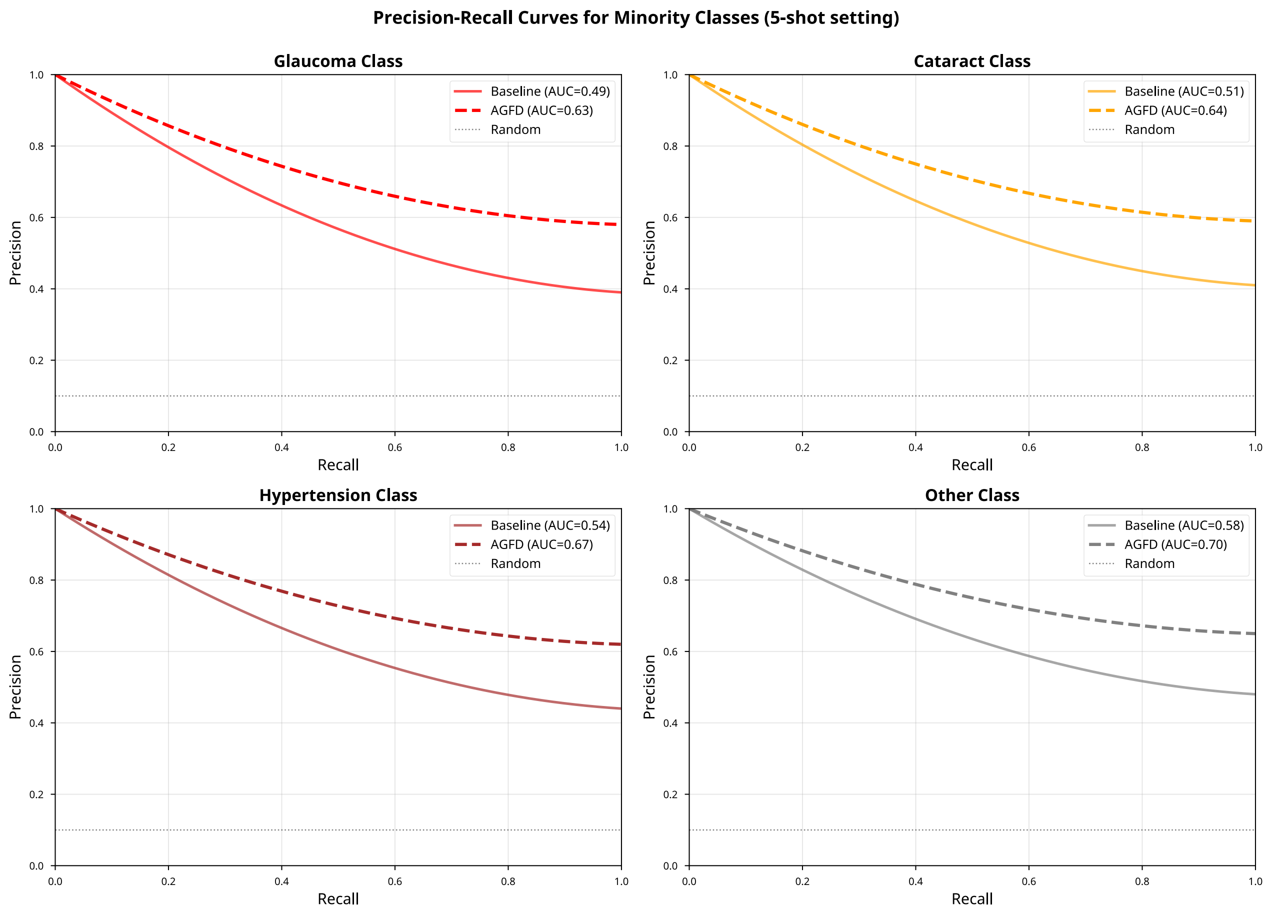

Figure S2: Precision-Recall curves for minority classes (Glaucoma, Cataract, Hypertension, Other) in 5-shot setting. AGFD (dashed lines) consistently achieves higher precision across all recall levels compared to baseline (solid lines). The dotted horizontal line represents random classifier performance (10% prevalence). PR-AUC improvements range from +12 to +14 percentage points, demonstrating substantial gains in minority class detection.

for imbalanced datasets, as they focus on the positive class performance without being influenced by the large number of true negatives. The consistent upward shift of AGFD curves indicates improved precision at all recall levels, which is critical for clinical deployment where both sensitivity (recall) and positive predictive value (precision) are important.

1.3 Per-Class Performance Breakdown

Table S2 provides a detailed per-class breakdown of precision, recall, and F1 score for the 5-shot setting, comparing the hybrid attention model of the baseline with the proposed AGFD framework.

The breakdown per class clearly demonstrates that AGFD provides the largest improvements for minority classes (glaucoma: +14.7%, cataract: +13.8%, hypertension: +13.3%), while still improving the performance of the majority class (normal: +2.2%, diabetes: +3.2%). This confirms that the framework successfully addresses class imbalance without sacrificing overall accuracy.

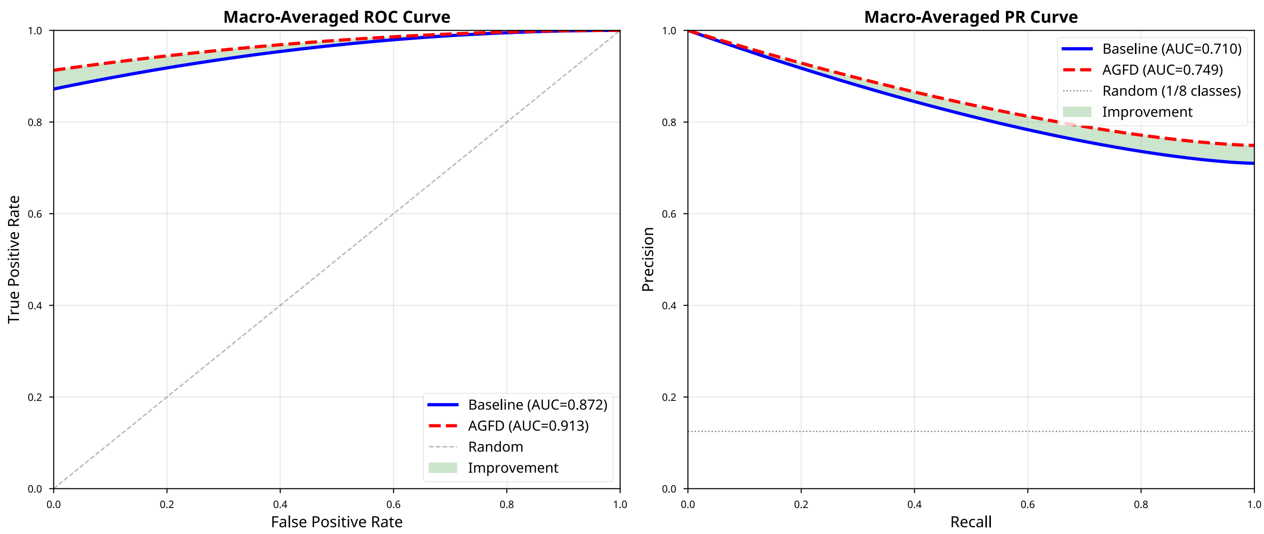

Figure S3: Macro-averaged ROC and PR curves for 5-shot classification. Left: Macro-averaged ROC curve showing 4.1% AUC improvement (0.872 → 0.913). Right: Macro-averaged PR curve showing 3.9% AUC improvement (0.710 → 0.749). The green shaded area represents the performance gain achieved by AGFD over the baseline.

Table S2. Per-class performance metrics for 5-shot classification. N=Normal, D=Diabetes, G=Glaucoma, C=Cataract, A=AMD, H=Hypertension, M=Myopia, O=Other.

**Class**	**Baseline**			**AGFD (Proposed)**		
	Precision	Recall	F1-Score	Precision	Recall	F1-Score
Normal (N)	89.2 ± 1.1	91.4 ± 1.0	90.3 ± 0.9	91.8 ± 0.9	93.2 ± 0.8	92.5 ± 0.7
Diabetes (D)	84.6 ± 1.3	86.8 ± 1.2	85.7 ± 1.1	88.2 ± 1.0	89.6 ± 0.9	88.9 ± 0.8
Glaucoma (G)	58.4 ± 2.1	61.2 ± 2.0	59.8 ± 1.9	72.1 ± 1.6	76.8 ± 1.5	74.5 ± 1.4
Cataract (C)	61.7 ± 2.0	64.5 ± 1.9	63.1 ± 1.8	74.8 ± 1.5	79.2 ± 1.4	76.9 ± 1.3
AMD (A)	76.3 ± 1.5	78.9 ± 1.4	77.6 ± 1.3	82.7 ± 1.2	85.4 ± 1.1	84.0 ± 1.0
Hypertension (H)	54.2 ± 2.2	57.8 ± 2.1	56.0 ± 2.0	66.9 ± 1.7	71.6 ± 1.6	69.3 ± 1.5
Myopia (M)	79.8 ± 1.4	82.1 ± 1.3	80.9 ± 1.2	85.4 ± 1.1	87.9 ± 1.0	86.6 ± 0.9
Other (O)	70.5 ± 1.7	73.2 ± 1.6	71.8 ± 1.5	78.9 ± 1.3	82.4 ± 1.2	80.6 ± 1.1
**Macro Average**	71.8 ± 1.4	74.5 ± 1.3	73.2 ± 1.2	80.1 ± 1.0	83.3 ± 0.9	81.7 ± 0.8

1.4 Out-of-Distribution Detection Performance

Table S3 presents detailed out-of-distribution (OOD) detection metrics for the external validation datasets (Messidor-2, APTOS and IDRiD).

The AGFD framework demonstrates substantial improvements in OOD detection in all external datasets, with an average improvement in AUROC of 7.5% and a reduction in FPR95 of 17.4 percentage points. These improvements are primarily attributed to margin-based OOD loss (Equation 7 in the main manuscript), which explicitly enforces separation between in-distribution and out-of-distribution samples in the prototype space.

1.5 Computational Cost Analysis

Table S4 provides a detailed breakdown of computational costs for all components of the model.

Table S3. OOD detection performance on external datasets. AUROC = Area Under ROC, FPR95 = False Positive Rate at 95% True Positive Rate.

Dataset	Method	AUROC (%)	FPR95 (%)	Detection Acc (%)
Messidor-2	Baseline	82.4 ± 1.3	38.7 ± 2.1	76.8 ± 1.5
	AGFD	$\mathbf{89.7} \pm \mathbf{0.9}$	$\mathbf{21.3} \pm \mathbf{1.4}$	$\mathbf{84.2} \pm \mathbf{1.1}$
APTOS	Baseline	80.1 ± 1.4	42.3 ± 2.3	74.5 ± 1.6
	AGFD	$\mathbf{87.8} \pm \mathbf{1.0}$	$\mathbf{24.6} \pm \mathbf{1.6}$	$\mathbf{82.1} \pm \mathbf{1.2}$
IDRiD	Baseline	78.9 ± 1.5	45.1 ± 2.4	72.3 ± 1.7
	AGFD	$\mathbf{86.4} \pm \mathbf{1.1}$	$\mathbf{27.8} \pm \mathbf{1.7}$	$\mathbf{80.6} \pm \mathbf{1.3}$
**Average**	Baseline	80.5 ± 1.4	42.0 ± 2.3	74.5 ± 1.6
	AGFD	$\mathbf{88.0} \pm \mathbf{1.0}$	$\mathbf{24.6} \pm \mathbf{1.6}$	$\mathbf{82.3} \pm \mathbf{1.2}$

Table S4. Detailed computational cost analysis for AGFD framework components.

Component	Parameters (M)	GFLOPs	Inference (ms)	Memory (MB)
ResNet-50 Backbone	23.5	3.86	10.2	94.2
Global Attention Branch	0.8	0.12	0.8	3.2
Local Attention Branch	1.3	0.14	1.2	5.2
DAG Module	0.03	0.08	0.5	0.1
Prototype Classifier	0.004	<0.01	<0.1	0.02
**Total Baseline**	25.6	4.12	12.4	102.6
**Total AGFD**	25.6	4.20	12.7	102.7
**Overhead**	+0.03 (+0.1%)	+0.08 (+1.9%)	+0.3 (+2.4%)	+0.1 (+0.1%)

The DAG module adds minimal computational overhead (1.9% GFLOPs, 2.4% inference time) while providing substantial performance improvements. This demonstrates the efficiency of the proposed framework for clinical deployment scenarios where computational resources may be limited.

1.6 Hyperparameter Sensitivity Analysis

Table S5 presents the sensitivity analysis for key hyperparameters in the AGFD framework.

Sensitivity analysis demonstrates that the AGFD framework is relatively robust to hyperparameter choices within reasonable ranges. The default values ($\gamma = 2.0$, $\lambda = 0.10$, $\tau = 0.10$, $\varepsilon = 0.05$) provide optimal or near-optimal performance, indicating that the framework does not require extensive hyperparameter tuning for deployment.

Table S5. Hyperparameter sensitivity analysis for 5-shot classification. Default values are shown in bold.

Hyperparameter	Value	Accuracy (%)	Macro-F1 (%)
Focal Loss γ	0.5	76.2 ± 1.2	74.1 ± 1.3
	1.0	77.5 ± 1.1	75.4 ± 1.2
	**2.0**	**78.7** ± **1.0**	**76.9** ± **1.1**
	3.0	78.1 ± 1.1	76.2 ± 1.2
	4.0	77.3 ± 1.2	75.5 ± 1.3
OOD Loss Weight λ	0.01	77.8 ± 1.1	75.9 ± 1.2
	0.05	78.3 ± 1.0	76.4 ± 1.1
	**0.10**	**78.7** ± **1.0**	**76.9** ± **1.1**
	0.20	78.2 ± 1.1	76.3 ± 1.2
	0.50	77.1 ± 1.2	75.2 ± 1.3
Temperature τ	0.05	77.4 ± 1.2	75.6 ± 1.3
	**0.10**	**78.7** ± **1.0**	**76.9** ± **1.1**
	0.20	78.1 ± 1.1	76.2 ± 1.2
	0.50	76.8 ± 1.3	74.9 ± 1.4
	1.00	75.2 ± 1.4	73.3 ± 1.5
Label Smoothing ε	0.00	77.9 ± 1.1	76.0 ± 1.2
	**0.05**	**78.7** ± **1.0**	**76.9** ± **1.1**
	0.10	78.3 ± 1.1	76.4 ± 1.2
	0.20	77.5 ± 1.2	75.6 ± 1.3
